# Multi-population genome-wide association study implicates immune and non-immune factors in pediatric steroid-sensitive nephrotic syndrome

**DOI:** 10.1038/s41467-023-37985-w

**Published:** 2023-04-29

**Authors:** Alexandra Barry, Michelle T. McNulty, Xiaoyuan Jia, Yask Gupta, Hanna Debiec, Yang Luo, China Nagano, Tomoko Horinouchi, Seulgi Jung, Manuela Colucci, Dina F. Ahram, Adele Mitrotti, Aditi Sinha, Nynke Teeninga, Gina Jin, Shirlee Shril, Gianluca Caridi, Monica Bodria, Tze Y. Lim, Rik Westland, Francesca Zanoni, Maddalena Marasa, Daniel Turudic, Mario Giordano, Loreto Gesualdo, Riccardo Magistroni, Isabella Pisani, Enrico Fiaccadori, Jana Reiterova, Silvio Maringhini, William Morello, Giovanni Montini, Patricia L. Weng, Francesco Scolari, Marijan Saraga, Velibor Tasic, Domenica Santoro, Joanna A. E. van Wijk, Danko Milošević, Yosuke Kawai, Krzysztof Kiryluk, Martin R. Pollak, Ali Gharavi, Fangmin Lin, Ana Cristina Simœs e Silva, Ruth J. F. Loos, Eimear E. Kenny, Michiel F. Schreuder, Aleksandra Zurowska, Claire Dossier, Gema Ariceta, Magdalena Drozynska-Duklas, Julien Hogan, Augustina Jankauskiene, Friedhelm Hildebrandt, Larisa Prikhodina, Kyuyoung Song, Arvind Bagga, Hae Cheong, Gian Marco Ghiggeri, Prayong Vachvanichsanong, Kandai Nozu, Dongwon Lee, Marina Vivarelli, Soumya Raychaudhuri, Katsushi Tokunaga, Simone Sanna-Cherchi, Pierre Ronco, Kazumoto Iijima, Matthew G. Sampson

**Affiliations:** 1 Division of Nephrology, Boston Children’s Hospital, Boston, MA USA; 2 Kidney Disease Initiative & Medical and Population Genetics Program, Broad Institute of MIT and Harvard, Cambridge, MA USA; 3 Genome Medical Science Project (Toyama), National Center for Global Health and Medicine (NCGM), Tokyo, Japan; 4 Department of Human Genetics, Graduate School of Medicine, The University of Tokyo, Tokyo, Japan; 5 Division of Nephrology, Department of Medicine, Columbia University College of Physicians and Surgeons, New York, NY USA; 6 Sorbonne Université, UPMC Paris 06, Institut National de la Santé et de la Recherde Médicale, Unité Mixte de Rechereche, S 1155 Paris, France; 7 Kennedy Institute of Rheumatology, University of Oxford, Roosevelt Drive, Headington, Oxford, OX3 7FY United Kingdom; 8 Center for Data Sciences, Brigham and Women’s Hospital, Harvard Medical School, Boston, MA USA; 9 Divisions of Genetics and Rheumatology, Department of Medicine, Brigham and Women’s Hospital, Harvard Medical School, Boston, MA USA; 10 Program in Medical and Population Genetics, Broad Institute of MIT and Harvard, Cambridge, MA USA; 11 Department of Pediatrics, Kobe University Graduate School of Medicine, Kobe, Japan; 12 Department of Biochemistry and Molecular Biology, University of Ulsan College of Medicine, Songpa-gu, Seoul Korea; 13 Renal Diseases Research Unit, Genetics and Rare Diseases Research Division, Istituto di Ricovero e Cura a Carattere Scientifico Ospedale Pediatrico Bambino Gesù, Rome, Italy; 14 Nephrology, Dialysis and Transplantation Unit, Department of Emergency and Organ Transplantation, University of Bari Aldo Moro, Bari, Italy; 15 Department of Pediatrics, AIIMS, New Delhi, India; 16 Department of Pediatric Nephrology, Amalia Children’s Hospital, Radboud University Medical Center, Nijmegen, The Netherlands; 17 Department of Medicine, Boston Children’s Hospital, Boston, MA USA; 18 Department of Pediatrics, Harvard Medical School, Boston, MA USA; 19 Laboratory on Molecular Nephrology, IRCCS Instituto Giannina Gaslini, Genoa, Italy; 20 Department of Nephrology and Renal Transplantation, IRCCS Instituto Giannina Gaslini, Genoa, Italy; 21 Department of Pediatric Nephrology, VU University Medical Center, Amsterdam, The Netherlands; 22 Division of Transplantation, Department of Surgery, University of Pennsylvania, Philadelphia, PA USA; 23 Department of Pediatric Nephrology, Dialysis and Transplantation, Clinical Hospital Hospital Center Zagreb, University of Zagreb Medical School, Zagreb, Croatia; 24 Division of Nephrology and Pediatric Dialysis, Bari Polyclinic Giovanni XXIII Children’s Hospital, Bari, Italy; 25 Department of Nephrology, Dialysis and Transplant Unit, University Hospital of Modena, Modena, Italy; 26 Surgical, Medical and Dental Department of Morphological Sciences, Section of Nephrology, University of Modena and Reggio Emilia, Modena, Italy; 27 Unità Operativa Nefrologia, Azienda Ospedaliero-Universitaria di Parma, Dipartimento di Medicina e Chirurgia, Università di Parma, Parma, Italy; 28 Department of Nephrology, Medicine and General University Hospital, Charles University, Prague, Czech Republic; 29 Department of Pediatrics, ISMETT, Palermo, Italy; 30 Pediatric Nephrology, Dialysis and Transplant Unit, Fondazione IRCCS Ca’ Granda-Ospedale Maggiore Policlinico, Milano, Italy; 31 Department of Clinical Sciences and Community Health, University of Milan, Milan, Italy; 32 Department of Pediatric Nephrology, UCLA Medical Center and UCLA Medical Center-Santa Monica, Los Angeles, CA USA; 33 Department of Medical and Surgical Specialties, Radiological Sciences, and Public Health, Division of Nephrology and Dialysis, University of Brescia and ASST Spedali Civili of Brescia, Brescia, Italy; 34 Department of Pediatrics, University of Split, Split, Croatia; 35 Department of Pediatric Nephrology, University Children’s Hospital, Skopje, Macedonia; 36 Division of Nephrology and Dialysis Unit, University of Messina, Sicily, Italy; 37 Croatian Academy of Medical Sciences, Praska 2/III p.p. 27, 10000 Zagreb, Croatia; 38 Division of Nephrology, Beth Israel Deaconess Medical Center, Boston, MA USA; 39 Department of Pediatric, Division of Pediatric Nephrology, Columbia University Irving Medical Center New York-Presbyterian Morgan Stanley Children’s Hospital in New York, New York, NY USA; 40 Department of Pediatrics, Interdisciplinary Laboratory of Medical Investigation, Faculty of Medicine, Federal University of Minas Gerais, Belo Horizonte, Brazil; 41 The Charles Bronfman Institute for Personalized Medicine, Icahn School of Medicine at Mount Sinai, New York, NY USA; 42 Institute for Genomic Health, Icahn School of Medicine at Mount Sinai, New York, NY USA; 43 Division of Genomic Medicine, Department of Medicine, Icahn School of Medicine at Mount Sinai, New York, NY USA; 44 Division of General Internal Medicine, Department of Medicine, Icahn School of Medicine at Mount Sinai, New York, NY USA; 45 Department of Pediatrics, Nephrology and Hypertension, Medical University Gdansk, Gdansk, Poland; 46 AP-HP, Pediatric Nephrology Department, Hôpital Robert-Debré, Paris, France; 47 Pediatric Nephrology, Hospital Universitari Vall d’Hebron, Universitat Autónoma de Barcelona, Barcelona, Spain; 48 Institute of Clinical Medicine, Faculty of Medicine, Vilnius University, Vilnius, Lithuania; 49 Research and Clinical Institute for Pediatrics, Pirogov Russian National Research Medical University, Taldomskava St, 2, Moscow, Russia; 50 Department of Pediatrics, Hallym University Sacred Heart Hospital, 22, Gwanpyeong-ro 170 beon-gil, Dongan-gu, Anyang-si, Gyeonggi-do 14068 Korea; 51 Department of Pediatrics, Faculty of Medicine, Prince of Songkla University, Hat-Yai, Songkhla 90110 Thailand; 52 Division of Nephrology, and Dialysis, Department of Pediatric Subspecialities, Istituto di Ricovero e Cura a Carattere Scientifico Ospedale Pediatrico Bambino Gesù, Rome, Italy; 53 Department of Biomedical Informatics, Harvard Medical School, Boston, MA USA; 54 Centre for Genetics and Genomics Versus Arthritis, University of Manchester, Manchester, UK; 55 Department of Nephrology, Centre Hospitalier du Mans, Le Mans, France; 56 Hyogo Prefectural Kobe Children’s Hospital, Kobe, Japan; 57 Department of Advanced Pediatric Medicine, Kobe University Graduate School of Medicine, Kobe, Japan; 58 Division of Renal Medicine, Department of Medicine, Brigham and Women’s Hospital, Harvard Medical School, Boston, MA USA

**Keywords:** Minimal change disease, Genetics, Paediatric kidney disease

## Abstract

Pediatric steroid-sensitive nephrotic syndrome (pSSNS) is the most common childhood glomerular disease. Previous genome-wide association studies (GWAS) identified a risk locus in the HLA Class II region and three additional independent risk loci. But the genetic architecture of pSSNS, and its genetically driven pathobiology, is largely unknown. Here, we conduct a multi-population GWAS meta-analysis in 38,463 participants (2440 cases). We then conduct conditional analyses and population specific GWAS. We discover twelve significant associations—eight from the multi-population meta-analysis (four novel), two from the multi-population conditional analysis (one novel), and two additional novel loci from the European meta-analysis. Fine-mapping implicates specific amino acid haplotypes in *HLA-DQA1* and *HLA-DQB1* driving the HLA Class II risk locus. Non-HLA loci colocalize with eQTLs of monocytes and numerous T-cell subsets in independent datasets. Colocalization with kidney eQTLs is lacking but overlap with kidney cell open chromatin suggests an uncharacterized disease mechanism in kidney cells. A polygenic risk score (PRS) associates with earlier disease onset. Altogether, these discoveries expand our knowledge of pSSNS genetic architecture across populations and provide cell-specific insights into its molecular drivers. Evaluating these associations in additional cohorts will refine our understanding of population specificity, heterogeneity, and clinical and molecular associations.

## Abstract (web summary)

Here, the authors have performed a multi-population GWAS meta-analysis of pediatric steroid sensitive nephrotic syndrome cases to discover 12 loci (4 novel), fine-map HLA, implicate kidney and immune factors, and associate the polygenic risk score with earlier disease onset.

## Introduction

Pediatric steroid-sensitive nephrotic syndrome (pSSNS) is a rare disease of the glomerular filtration barrier. Its incidence ranges from 1.15–16.9 cases in every 100,000 children, occurring most frequently in South Asian populations^1^. pSSNS causes massive proteinuria and increases the risk of thromboembolism, sepsis, and progression to chronic kidney disease (CKD)/end-stage kidney disease (ESKD)^2–7^. And those progressing to ESKD have increased odds of recurrent NS in their allograft^8^. pSSNS is impactful across the lifespan—31–50% of those affected have relapses in adulthood^9^. Much of pSSNS’s morbidity is related to side effects of the non-specific immunosuppressants which allow some to achieve remission of their proteinuria^7, 10–17^.

Despite intensive investigation, there are no known monogenic forms of pSSNS to illuminate its pathobiology. However, we know that immune dysregulation is a major contributor^18,19^. But determining causal immune factors via case-control studies of cytokines profiles, cell types, and transcriptomic signatures is challenging. The dynamic responses of the immune system at different disease stages and to various stimuli make it difficult to determine whether observed differences are causal, correlated, or due to independent biological/environmental factors. And kidney tissue in children is rarely available to determine intrarenal, molecular drivers of pSSNS.

Previous GWAS have discovered four pSSNS risk loci^20–24^. In each GWAS, the top risk locus is in the HLA Class II region. Two other loci are plausibly immune-related, with the closest genes being Calcium Homeostasis Modulator Family Member 6 (*CALHM6*)^25^ and TNF Superfamily Member 15 (*TNFSF15)*^26^. The lead SNP of the fourth locus is within nephrin (*NPHS1)*, a fundamental glomerular gene implicated in Mendelian NS^27^. These studies are illuminating but limited by smaller sample sizes, primarily population-specific analyses, and limited post-GWAS analysis. Here we conducted a large and diverse GWAS of pSSNS to discover and more fully characterize disease-associated genetic variation and unravel its pathogenesis at the interface of the immune system and kidney.

## Results

**Fig. 1:**
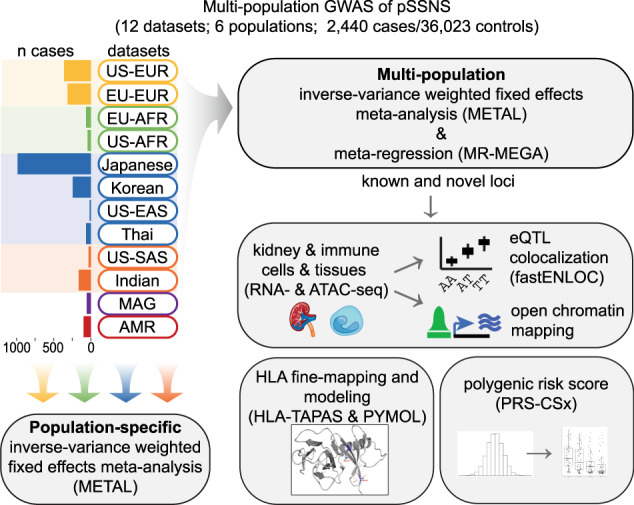
Flowchart of study design. 12 datasets across six populations were used for population-specific and multi-population GWAS meta-analyses. The population assignment and number of cases for each dataset are indicated (yellow=European (EUR), green=African (AFR), blue=East Asian (EAS), orange=South Asian (SAS), purple=Maghrebian (MAG), red=Admixed American (AMR)). Post-GWAS analyses include colocalization with both kidney and immune eQTL datasets and overlap of SNPs within credible sets with single-cell kidney and immune open chromatin (ATAC-seq). HLA imputation with HLA-TAPAS was used to identify classical alleles and specific amino acids associated with pSSNS, followed by modeling of the HLA protein and stability predictions. Dataset summary statistics were used to generate polygenic risk scores using PRS-CSx and associations with clinical covariates were tested. pSSNS= pediatric steroid-sensitive nephrotic syndrome, eQTL = expression quantitative trait loci.

**Fig. 2:**
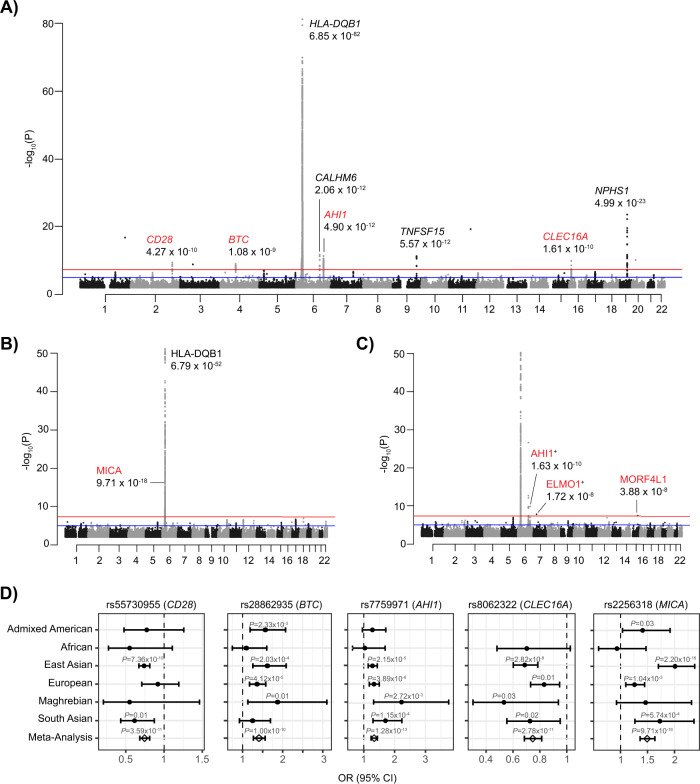
GWAS results. All loci are labeled by nearest gene with novel associations in red. **A** Multi-population meta-analysis of 2440 cases vs. 36,023 controls. The *P* value from test of deviance of full meta-regression model compared to the null model using MR-MEGA. **B** Multi-population conditional meta-analysis. The *P* value from multiple linear regression with COJO. **C** European meta-analysis of 674 cases vs. 6817 controls. Discoveries that included the summary statistics from suggestive SNPs available from Dufek et al. are indicated with + and only novel associations are labeled. The *P* values are from meta-analysis with METAL. **D** Multi-population and single-population odds ratios with 95% confidence interval for novel multi-population significant SNPs. The *P* value for MICA is from the conditional analysis with COJO, Maghrebian, and Admixed American *P* values are from logistic regression, and the rest are from inverse-variance fixed-effects meta-analysis with METAL. All *P* values in **A**–**D** are unadjusted for multiple testing and all tests are two-sided. Number of cases in each analysis: Admixed American *n* = 98, African *n* = 109, East Asian *n* = 1311, European *n* = 674, Maghrebian *n* = 55, South Asian *n* = 193, Meta-Analysis *n* = 2440.

We conducted a multi-population, fixed-effect, inverse-variance, meta-analysis across twelve GWAS datasets comprised of 2440 cases and 36,023 controls of Admixed American, African, East Asian, European, Maghrebian, and South Asian populations (Fig. 1, Supp. Fig. 1, Supp. Table 1).To account for population-driven effect heterogeneity, we also performed a meta-regression with MR-MEGA^28^. Given the increased power in the presence of heterogeneity across populations, we identified significant loci using MR-MEGA results. Eight loci (four new, and all outside HLA) were significant (MR-MEGA *p* < 5 × 10^−^^8^) (Table 1, Fig. 2A, Supp. Fig. 2). The lead SNPs of the novel loci were all intronic: (1) rs7759971 in Abelson Helper Integration Site 1*(AHI1*; *p* = 4.90 × 10^−^^12^); (2) rs55730955 in CD28 molecule (*CD28*; *p* = 4.27 × 10^−^^10^); (3) rs8062322 in C-type Lectin Domain Containing 16 A **(***CLEC16A*; *p* = 1.61 × 10^−^^10^); (4) rs28862935 in betacellulin **(***BTC*; *p* = 1.08 × 10^−^^9^). The remaining three significant loci located outside of the HLA region were previously reported^23,24^. The associations found near *NPHS1* and *TNFSF15* are driven by overlapping samples from Jia et al; however, this is an independent replication of the *CALHM6* locus. After conditioning on the lead SNPs, two more significant loci emerged: (5) rs1794497 upstream of *HLA-DQB1*, (*p* = 6.79 × 10^−^^52^); (6) rs2256318 in an intron of MHC Class I Chain-related Gene A (*MICA;* p = 9.70 × 10^−^^18^) (Fig. 2B, Supp. Fig. 3). Population-specific GWAS meta-analysis discovered two additional significant loci in Europeans (Fig. 2C, Supp. Table 2-3, Supp. Fig. 4): The lead SNPs were in introns of (7) rs111796602 in an intron of Engulfment and Cell Motility 1 (*ELMO1;* p = 1.72 × 10^−^^8^) and (8) rs12911841 in an intron of Mortality Factor 4 Like 1 (*MORF4L1*; *p* = 3.88 × 10^−^^8^). Loci with population-driven heterogeneity were observed at three loci. Variants at the *CALHM6* locus were associated with an increased risk in Europeans and those at the *TNFSF15* and *NPHS1* loci were associated with an increased risk in East Asians (Supp. Fig. 5, Supp. Fig. 6). The remaining loci showed similar effects across populations (Fig. 2D). Finally, there were 20 novel suggestive loci (MR-MEGA *p* < 1 × 10^−^^5^) in the multi-population GWAS (Supp. Table 4, Supp. Table 5). On a liability scale and excluding HLA, European heritability was 0.04 [CI: −0.08, 0.16] and East Asian heritability was 0.12 [CI: 0.04, 0.21], with large confidence intervals likely due to small effective sample sizes.

**Table 1:** Genome-wide significant SNPs from multi-population meta-analysis

					Fixed-effects meta-analysis			Meta-regression		
Nearest gene	Top SNP	Position (hg19)	EA	NEA	OR [95% CI]	*P* value	Het *χ*^2^ *P* value	*P* value	Population Het *P* value	Residual Het *P* value
Discovery meta-analysis										
*HLA-DQB1*	rs1063355	6:32627714	T	G	0.46 [0.42, 0.50]	4.51 × 10^−^^81^	0.09	6.85 × 10^−^^82^	0.26	0.07
*NPHS1*	rs412175^b^	19:36342103	C	T	1.65 [1.49, 1.83]	4.15 × 10^−^^22^	0.02	2.30 × 10^−^^24^	2.43 × 10^−^^4^	0.87
*CALHM6*	rs2637678^c^	6:116787378	C	T	0.79 [0.73, 0.85]	1.48 × 10^−10^	7.30 × 10^−^^5^	2.06 × 10^−^^12^	4.99 × 10^−^^4^	5.24 × 10^−3^
AHI1*	rs7759971	6:135746884	T	C	1.34 [1.24, 1.44]	1.28 × 10^−13^	0.52	4.90 × 10^−^^12^	0.74	0.30
*TNFSF15*	rs10817678	9:117579457	G	A	0.79 [0.73, 0.85]	2.92 × 10^−10^	0.04	5.57 × 10^−^^12^	7.76 × 10^−^^4^	0.82
CLEC16A*	rs8062322	16:11092319	A	C	0.75 [0.68, 0.81]	2.78 × 10^−11^	0.33	1.61 × 10^−^^10^	0.16	0.43
CD28*	rs55730955	2:204585956	A	T	0.74 [0.68, 0.81]	3.59 × 10^−11^	0.62	4.27 × 10^−^^10^	0.34	0.59
BTC*	rs28862935	4:75693465	A	G	1.41 [1.27, 1.56]	1.00 × 10^−^^10^	0.18	1.08 × 10^−^^09^	0.24	0.18
Conditional meta-analysis										
*HLA-DQB1*	rs1794497^d^	6:32649180	C	T	2.04 [1.89, 2.27]	6.79 × 10^−^^52^	0.03	-	-	'-
MICA*	rs2256318^d^	6:31381519	A	G	1.49 [1.36, 1.64]	9.71 × 10^−^^18^	4.18 × 10^−^^4^	-	-	-

The fixed-effect meta-analysis was performed with METAL. Its *P* value is from a two-sided inverse-variance weighted meta-analysis and the Het *χ*^2^ *P* value is from a two-sided chi-square test for heterogeneity. The meta-regression was performed with MR-MEGA, and all tests are two-sided and approximated by a chi-square distribution. The MR-MEGA *P* value tests the association of the SNP and is from test of deviance of full meta-regression model compared to the null model. Its population Het *P* value measures the heterogeneity in allelic effects that are correlated with GWAS populations and is from a test of deviance of the full model compared to the model excluding population principal components. The residual heterogeneity is the deviance of the full model. Conditional analysis was performed on all loci from the discover meta-analysis with exception of rs412175 and rs2637678. rs56117924 and rs2637681 were used in the conditional analysis, respectively. rs2256318 and rs1794497 are ~1.3 Mb apart with *r*^2^ < 0.13 across all the 1000 Genomes Project populations, and with an *r*^2^ = 0.04 when combining all the 1000 Genomes samples. MR-MEGA results are not available for conditional analysis. Novel loci *, EA effect allele, NEA non-effect allele, OR [95% CI] = Odds ratio with 95% confidence interval.

A number of insights emerged from evaluating disease associations, functions, and expression patterns of the lead SNPs and/or the closest genes at the novel non-HLA loci. First, PheWAS using Open Target Genetics (http://genetics.opentargets.org)^29^ found that SNPs at most loci were associated with diverse white blood cell traits, atopic disorders, and autoimmune conditions. For example, among the strongest associations with the lead SNPs at the following loci include: *CLEC16A, CD28, MICA*, and *ELMO1* with eosinophil counts; *AHI1* with monocyte and neutrophil counts, asthma, and hay fever (also shared by *CD28*); and *MICA* with type 1 diabetes.

Second, while most of these genes are primarily known for their role in immunity, many also have known roles in kidney diseases and cells. Common *AHI1* variants are associated with atopy, lupus, and diverse immune cell traits^29^. But rare, pathogenic *AHI1* coding variants cause the monogenic ciliopathy Joubert Syndrome, which includes cystic kidney disease^30^. *ELMO1* participates in Rac1 pathway activation and actin cytoskeletal rearrangement^31^, is expressed in podocytes^32^, and is associated with diabetic nephropathy^33^. *CD28*, a T-cell glycoprotein, binds a co-stimulatory molecule B7-1 (CD80) on antigen-presenting cells. B7-1 is expressed on human podocytes in some forms of nephrotic syndrome and blocking the B7-1/CD28 interaction with a CTLA-4 immunoglobulin can ameliorate proteinuria^34^. *MICA* is expressed in kidney endothelium, binds and activates cytotoxic CD8+T cells and NK cells, and has increased glomerular expression in lupus^35^. *BTC* contributes to inflammation by binding to epidermal growth factor receptor^36^, a gene whose kidney expression is upregulated following kidney injury^37^. *CLEC16A* takes part in the B cell receptor-dependent HLA-II pathway in human B cells^38^ but is also significantly expressed in the human podocytes (https://atlas.kpmp.org). *CLEC16A* is also involved in autophagy, mitophagy, and endolysosomal trafficking in multiple cell types^39,40^. Furthermore, it is also in close proximity to *CIITA*, a master transcription factor of HLA class II genes^41^ and Dexamethasone Inducible Transcript (*DEXI)*, a glucocorticoid-induced gene^42^.

We next turned to discovering specific variants and genes driving these GWAS loci and discerning whether they are acting in immune cells, kidney cells, or both.

**Fig. 3:**
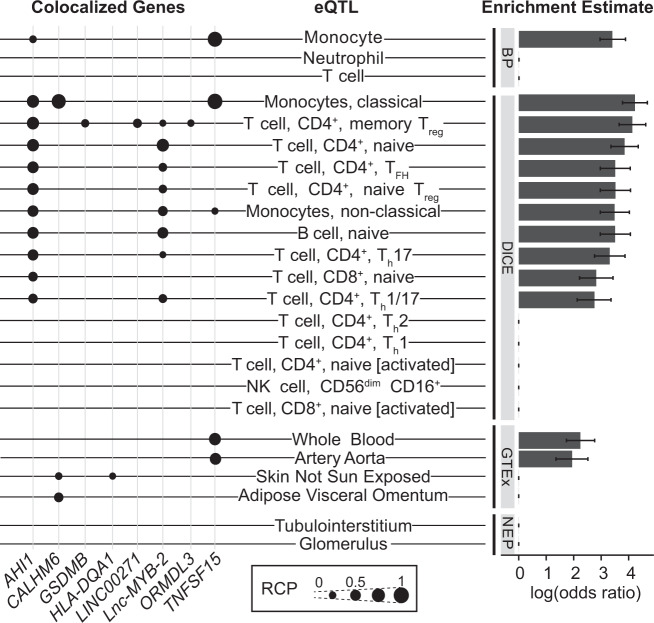
Colocalization of SSNS GWAS and eQTL datasets. Each eQTL dataset is labeled with colocalized loci (left) and enrichment estimates (right). The source of each eQTL dataset is labeled on vertical gray bars. BP BLUEPRINT, NEP NEPTUNE. Genes with regional colocalization probability (RCP) > 0.2 in at least one tissue/cell are included. pSSNS GWAS loci that colocalized with tissue/cell-type eQTLs are indicated by black dots, with larger dots indicating higher RCP. GTEx tissues without associations are excluded from this figure (see Supp. Fig. 7). Enrichment estimates from fastENLOC are based on genome-wide summary statistics from GWAS and include a shrinkage parameter that results in 0 enrichment for multiple tissues/cell types. Estimates are presented as the logarithm of the odds ratio ± standard error. logOR = 2 ~ OR = 7.5, logOR=3 ~OR = 20.1, logOR = 4 ~ OR = 54.6. eQTL sample sizes: NEPTUNE glomerulus *n* = 240, tubulointerstitial *n* = 311, BLUEPRINT *n* = 200 DICE *n* = 91.

First, we conducted colocalization with eQTL data from two functionally distinct kidney compartments (glomerulus and tubulointerstitium; NEPTUNE^43^), multiple tissues from GTEx^44^, and immune cells of healthy adults from DICE^45^ and BLUEPRINT^46^. Overall, pSSNS GWAS SNPs demonstrated significant enrichment in multiple immune cell eQTLs, led by a 69× and 62× increased odds of being monocyte and CD4+memory Treg eQTLs, respectively (Fig. 3). On an individual gene level, seven genes colocalized with immune cell eQTLs (Fig. 3, Supp. Table 6). Three genes were closest to the lead GWAS SNP at their respective locus—*CALHM6*, *AHI1*, and *TNFSF15*. Each were significantly colocalized with monocyte eQTLs. *AHI1* also colocalized with many T-cell subsets and naive B cells. Finally, a suggestive locus on chromosome 17 colocalized in CD4+memory Treg cells with two distinct genes—Gasdermin B (*GSDMB)* and ORMDL sphingolipid biosynthesis regulator 3 (*ORMDL3*). The *GSDMB/ORMDL3* locus is associated with multiple autoimmune disorders and eosinophilic inflammation-driven asthma^47^. In asthma, higher *GSDMB* expression is correlated with increased interferon signaling and MHC class I antigen presentation^48^. Notably, there was no colocalization with kidney eQTLs despite sufficient sample sizes (Supp. Fig. 7).

We then created a 95% credible set for all non-HLA significant loci and assessed their overlap with ATAC-seq derived open chromatin data from immune^49^ and kidney cells^50, 51^ (Supp. Table 7). The SNPs with the highest posterior inclusion probability (PIP) for *AHI1*, rs7759971 (PIP = 0.40), overlapped with open chromatin of multiple immune cell types, including CD34 + cells, common lymphoid and myeloid progenitors, hematopoietic stem cells, and multipotent progenitors. Similarly, the top SNP for CD28, rs55730955 (PIP = 0.68), overlapped with CD4+and CD8+open chromatin. The top PIP SNP for *BTC*, *CLEC16A,* and *TNFSF15* had no overlap with open chromatin. However, each locus had individual SNPs with lower PIPs that overlapped with both immune and kidney cell open chromatin.

We next fine-mapped the HLA risk locus to discover classical HLA alleles and amino acids associated with pSSNS (Supp. Table 8). We first imputed across the extended MHC region using a multi-population HLA imputation panel^52^, resulting in 640 classical HLA alleles, 4513 amino acids in HLA proteins, and 49,321 SNPs in the extended MHC region for association. We used population-specific and multi-population SNP-level logistic regression, to identify specific SNPs and classical alleles associated with pSSNS (Supp. Table 9, Supp. Note 1, Supp. Table 10, Supp. Fig. 8).

**Fig. 4:**
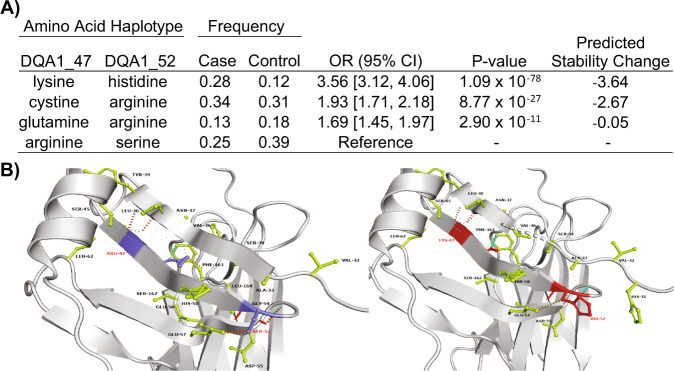
*HLA-DQA1* amino-acid associations and stability prediction. **A** Increased risk and predicted stability change of the two-amino-acid residue haplotypes at *HLA-DQA1* positions 47 and 52. Odds ratios and *P* values (two-sided) are from a joint logistic regression with arginine_47_-serine_52_, the most common, set as reference, adjusting for population-specific principal components and continental populations. The reference haplotype confers the strongest protection (i.e., odds ratios indicate increase in risk compared to arginine_47_-serine_52_). Decreasing values of the predicted stability change indicate decreasing stability. **B** Protein structure for the reference haplotype arginine_47_-serine_52_ (left, blue) and lysine_47_-histidine_52_ (right, red). The residues in green display a potential interacting amino acid with mutated amino acids. The color scheme for interactions (dashed lines) is as follows: cyan for Van der Waals [VDW], red for hydrogen bonds, green for hydrophobic bonds, sky blue for carbonyl bonds, and orange for polar bonds. Amino acids displayed with no visible bonds indicate a prediction of weak VDW bonds.

We next turned to discovering specific HLA amino-acid positions most associated with risk of pSSNS through logistic regression analysis of all residues at each position. Amino-acid position 47 in *HLA-DQA1* was most strongly associated with pSSNS (*P*_omnibus_ = 7.73 × 10^−^^83^) (Supp. Table 11, Supp. Fig. 9). Arginine was the most frequent amino-acid; a substitution to lysine conferred the greatest disease risk (*p* = 5.70 × 10^−^^80^; OR [95% CI] = 3.62 [3.17—4.14]). A second association in near-perfect linkage disequilibrium was identified at *HLA-DQA1* position 52 (*p* = 1.14 × 10^−^^82^). Arginine was again the most common amino-acid at this position, and a substitution to serine conferred the greatest protection from risk (*p* = 1.00 × 10^−^^28^; OR = 0.53 [0.47—0.59]). After conditioning, an independent association was discovered at *HLA-DQB1* position 26 (*p* = 3.22 × 10^−^^13^). A change from the most common amino-acid leucine to glycine conferred the most significant protection (*p* = 4.75 × 10^−^^12^; OR = 0.64 [0.60—0.73]). A haplotype analysis identified the 47_lysine_−52_histidine_ haplotype was associated with greatest increase in odds of pSSNS (Fig. 4A). *HLA-DQA1* position 47 is located on the outside of the peptide-binding groove and acts as a regulator of binding stability, which, when altered, has been suggested to mediate the development of autoimmune disorders^53^. Arginine at *HLA-DQA1* position 52 has been associated with autoimmune disorders, including type 1 diabetes^54^.

We then used DynaMut2^55^ to model the impact of the 47_lysine_−52_histidine_ haplotypes on protein structural stability (Fig. 4B). This is quantified by Delta Delta G (ddG), where ddG <0 predicts unstable structure. The haplotype consisting of lysine (47) and histidine (52) predicted the most instability (ddG = −3.64). Notably, the predicted increase in protein instability and increased odds of disease for each haplotype were concordant. This suggests pSSNS-associated haplotypes increase the odds of disease by increasing the instability of *HLA-DQA1* and altering its ability to properly form a stable HLA-II molecule.

Finally, we generated a multi-population pSSNS polygenic risk score (PRS) using summary statistics of 1974 cases and 20,039 controls from European, East Asian, African, and South Asian populations. We tested the association of the PRS with demographic and clinical phenotypes in 233 European children with sufficient clinical data from the EU-European sub-cohort, adjusting for four genetic principal components. The highest PRS quartile had significantly lower age of onset (Q4: 4.9 years) compared to the lowest quartile (Q1: 6.9 years, *p* = 2.79 × 10^−3^, Supp. Table 12). We did not find a significant association between PRS and sex or relapse pattern. Of note, we found concordant results using a PRS generated using discovery GWAS from a PRS score generated from European GWAS only (Supp. Table 12, Methods).

## Discussion

A number of important discoveries emerged from this study. First, we identified seven novel pSSNS loci—four novel loci from our multi-population meta-analysis (*BTC*, *AHI1*, *CD28,* and *CLEC16*), one locus from the corresponding conditional analysis (*MICA*), and two additional novel loci unique to the European meta-analysis (*ELMO1*, *MORFL1*). Second, we found that while the immunological connections with the lead SNPs and closest genes in these newly discovered loci are well-established, most of them also have a bona fide, but overall less understood role, in kidney cells and diseases. Identifying the genes, cells, and organ systems with which each of these identified risk loci act will be an important future step.

For instance, is the lead SNP near *AHI1* in fact altering the function of *AHI1* itself? And if so, how will we come to understand how rare, coding variants in this gene cause a structural, cystic kidney disease while a common, non-coding variant impacting the same gene contributes to an immunologically-mediated, acquired condition of the kidney? The availability of single-cell omics data from larger samples sizes and pediatric kidney tissue will be critical to help sort this out. These new datasets will also help post-GWAS studies like colocalization with eQTL and open chromatin, as we hypothesize that the paucity of kidney eQTLs we observed may be due to mapping pSSNS GWAS data to molecular datasets that do not adequately represent rare kidney cell types or changes that occur in the childhood age. In another example, we have now identified a two amino-acid haplotype in *HLA-DQA1* that increases risk of pSSNS. By what mechanism is it doing this?

Third, while our field has been long-focused on the role of T cells in pSNNS and more recently B cells, our results now suggest that monocyte and eosinophil gene dysregulation may also be a potential contributor to the pathogenesis of pSSNS. Are we observing a pathologic signature from resident monocytes and/or circulating monocytes. Alternatively, are some single-cell immune eQTLs a proxy for certain kidney subtypes that we don’t observe in our current bulk-level analysis? While SSNS is sometimes observed in the hypereosinophilic condition Kimura disease^56^, its etiology unknown. Defining the mechanism by which genetically driven changes in these cell types contribute to pSSNS onset is an important area of future inquiry.

Our analysis revealed a lack of significant colocalization between genetic variants associated with pSSNS and those associated with gene expression in kidney tissues. This could be consistent with the understanding that pSSNS is primarily a disease of immune dysregulation, with the kidney being the end organ affected. It is thus plausible that most of the genetic risk for pSSNS is mediated through genes and pathways in immune cells. This is supported by previous research that has shown that transplanting healthy kidneys into patients with a history of NS can result in recurrence of disease. Furthermore, a case report described that retransplanting the now-diseased kidney into a patient without NS resulted in disease cessation in the transplanted kidney, suggesting that an abnormal immune system alone is sufficient to cause pSSNS^8,57^.

Nevertheless, we posit that, as exemplified by the NPHS1 risk locus, there may be genetic factors that contribute to pSSNS through regulatory effects on kidney cells. Larger GWAS and eQTL studies will provide greater statistical power, which may uncover colocalized variants in the kidney. In addition, eQTL analysis using single-cell RNA-seq should provide more power to detect eQTLs in rare cell types, such as podocytes and glomerular endothelium. These signals that may be currently missed with the bulk methods. We may also more readily detect kidney cell impact from these pediatric SSNS GWAS data if use kidney cell omics datasets derived solely from children. Integrating pediatric-derived GWAS and molecular data (e.g. eQTL, open chromatin) derived by patients of the same age may capture state-dependent relationships that are obscured when using adult omics data with pediatric GWAS summary statistics. Finally, it is important to note that regulation of genes in kidney cells by risk SNPs may occur through mechanisms that are not linked to levels of gene expression, such as allele-specific expression of *NPHS1*^23^. Therefore, future studies should also consider looking for evidence of allele-specific expression, splice QTLs, protein QTLs, or other forms of dysregulation to uncover the impact of GWAS alleles on kidney cells.

Fourth, we discovered specific amino-acid changes in *HLA-DQA1* and *-DQB1* associated with pSSNS that should empower subsequent studies to illuminate pathomechanisms at the risk locus that has been identified in every GWAS of pSSNS to date. But change in *HLA-DQA1* and *HLA-DQB1* gene expression due to genetic variation in the MHC region has also been implicated in association with eGFR, a common complex kidney trait^28,58^. How is altered expression of these genes involved in both a rare glomerular disease and a common, complex kidney trait? We don’t currently know. Ultimately, applying in silico methods to high-quality, human-derived immune- and kidney-omics datasets should help pare down candidate alleles, genes, cell types, and mechanisms to a manageable number for subsequent experimental studies in cells and model systems.

Finally, the association of higher PRS with younger age of onset suggests that a stronger genetic predisposition to disease lowers the threshold of an individual to develop pSSNS in the context of environmental factors and may ultimately help share clinical screening and care. We must now evaluate this PRS in other cohorts, such as in cohorts of pSSNS from other global populations, adults with the disease, or children with other forms of childhood-onset NS. Studying the molecular correlates of a high PRS score could also shed light on pathobiology driven by an increased burden of genetic risk of this disease.

There are limitations to this study. Cases and the reference population were not all genotyped on the same SNP array. While we used a robust strategy to account for the use of different SNPs arrays containing different SNPs, this still adds heterogeneity. Heterogeneity is also added by the age of onset pSSNS not being identical for all groups, with the Columbia-originating cohort including patients less than 21 as opposed to 18 for the other groups. And for the most part, we did not recruit healthy controls. Rather, we relied on available reference populations, assuming that, as a rare disease, pSSNS cases were absent within them. Each of these factors would be predicted to reduce power for discovery. Our ability to accurately measure heritability is limited. This is because sparse signals with strong effects can lead to less efficient estimates and low sample sizes can downwardly bias the results^59^. Moreover, heritability can vary among populations. Thus, to better understand heritability of pSSNS within and across populations, it is crucial to increase sample sizes across all populations. Finally, the sample sizes for South Asian, Maghrebian, and African could result in overfitting of summary statistics and were too low to allow us to perform rigorous post-GWAS analysis of their results on a population-specific basis. Our novel genome-wide significant loci revealed consistent directionality across all populations, but with varying magnitudes and significance. Obtaining independent datasets with sufficient sample sizes from each population will enhance our understanding of population heterogeneity and refine estimates of effect size.

In conclusion, the discoveries emerging from our global GWAS of pSSNS expand our knowledge of the genetic architecture of this disease and accelerate our understanding of its molecular underpinnings and clinical implications.

## Methods

This research was conducted with the informed consent of all study participants and had ethical approval from the Boston Children’s Hospital IRB.

Figures were generated with R (v3.6.3) and ggplot2 (v3.3.5).

### GWAS data summary

Recruitment of samples and statistical analyses varied by recruiting group. Details for each dataset are described below and in Supp. Table 1.

#### GWAS data from NEPHROVIR/EU

Sample collection and genotype calling were done at Sorbonne Université in Paris. Pediatric steroid-sensitive nephrotic syndrome was defined as proteinuria >0.25 g/mmol, serum albumin <25 g/L (< 30 in France), full response within four weeks of 60 mg/m^2^/day of oral prednisone or prednisolone, and age of onset <18 years old. 244 previously reported European patients from the NEPHROVIR study^21^ were combined with 159 newly recruited European patients recruited from France, Lithuania, Poland, Russia, Italy. Healthy adult controls (*n* = 300) were recruited from Lyon, France, and combined with population-matched controls from the 1000 Genomes Project Phase 3 release (*n* = 493)^60^ and the 3Cites Cohort (*n* = 2000). There were also 56 sub-Saharan African cases with 451 African controls from the 1000 Genomes Project and 85 Maghrebian cases with 261 Moroccan population-matched controls. Both were reanalyzed from a previous report^21^. There were 160 Indian cases with 93 population-matched controls. Samples were genotyped on the Illumina Human OmniExpress or Illumina Omni 2.5 arrays.

#### GWAS data from Columbia University (US Cohorts)

Sample collection and genotype calling was performed at Columbia University in New York. Cases were defined by local recruitment centers across the US, Europe, and Brazil as either minimal change disease or non-biopsied SSNS with age of disease onset <21. Five cohorts from Columbia University consisted of patients from European (*n*_cases_=371, *n*_controls_ = 4359), East Asian (*n*_cases_ = 17, *n*_controls_ = 443), sub-Saharan African (*n*_cases_ = 65, *n*_controls_ = 7344), South Asian (*n*_cases_ =39, *n*_controls_ = 534) and Admixed American (*n*_cases_ = 109, *n*_controls_ = 13,266) populations. As defined by the 1000 Genomes Project, Admixed Americans (AMR) include the following populations: Puerto Rican in Puerto Rico (PUR), Columbian in Medellin (CLM), Peruvian in Lima (PEL), Mexican Ancestry in Los Angeles (MXL). The genotyping of the cases used multiple versions of MEGA (Multi-Ethnic Global Array) chips that includes MEGA 1.0, MEGA 1.1, and MEGA^EX^. The controls that were genotyped on MEGA 1.0 were downloaded from NCBI dbGAP (IDAT files) from the PAGE consortium^61^. The differences between the chips were corrected first by mapping all the SNPs to a common cluster file in Genome Studio for individual MEGA platforms and then using Snpflip software.

#### GWAS data from Kobe University

Pediatric steroid-sensitive nephrotic syndrome cases were defined as urine protein to creatinine ratio ≥2.0, serum albumin ≤2.5 g/dl, and complete remission with 4–6 weeks after starting 60 mg/m1^2^ oral prednisolone per day and age of onset <18 years old. Three GWAS studies of SSNS in Japanese (*n*_cases_ = 987, *n*_controls_ = 3206), Korean (*n*_cases_ = 243, *n*_controls_ = 4041) and Thai (*n*_cases_ = 65, *n*_controls_ = 94) population were completed at The University of Tokyo, Japan. The Japanese GWAS data have been previously reported^22,23^. The Thai dataset was genotyped with the Axiom array. The Korean data was genotyped with the Affymetrix Axiom array for cases and Illumina OmniQuad chip for controls.

### Dataset QC, imputation, and GWAS

Quality control, imputation, and GWAS were conducted separately for each study location and population. GC lambda (GC_λ_) was used to assess inflation in all studies. The final case and control sizes and the number of variants tested can be found in Supp. Table 1 and Supp. Fig. 1. Supplementary Fig. 11 shows matching of cases and controls in PCA plots. Manhattan plots and GC can be found in Supp. Fig. 12 and genome-wide significant hits resulting from dataset GWAS are in Supp. Table 13.

#### GWAS data from NEPHROVIR/EU: EU-European, EU-African, Maghrebian, Indian

Each file was quality controlled separately to remove related individuals (IBD > 0.1875), low call rate (genotype rate <98%), and cases with discordant sex. SNPs were quality controlled for allele frequency (MAF < 0.01), call rate (genotype rate <98%) in all cohorts, and Hardy Weinberg equilibrium (HWE *P* < 1 × 10^−5^) in controls only. The EU-European datasets were generated in multiple files and were merged stepwise on the common subset of SNPs, with the previous QC procedure reapplied after each merge. PCA plots were constructed from PLINK v1.9 to identify population outliers and check for batch effects^62^. Pre-imputation QC was conducted using McCarthy Tools v4.3 with the TOPMed reference panel to check strand alignment and allele assignment. Insertions and deletions were excluded prior to imputation. Each population was imputed separately with cases and controls were imputed together on the TOPMed Imputation Server with the TOPMed r2 reference panel^63–65^. The QC was repeated after imputation and SNPs with low imputation quality (rsq <0.3) were excluded. After imputation, UCSC Liftover^66^ was used to convert SNP positions from each population dataset to build GRCh37 to match the build of summary statistics from other analyses. The association tests were completed using PLINK v1.9 under an additive model with principal component adjustment to account for population stratification.

#### GWAS data from Columbia University (US Cohorts): US-European, US-African, US-South Asian, US-East Asian, and Admixed American

Population was assigned by KING^67^ kinship analysis software and based on continental population as defined by the 1000 Genomes Project for all cases and controls^68,69^. Within each continental population (EUR, AFR, AMR, SAS, and EAS), we removed variants with genotype rate <99%, MAF < 0.01, and HWE *P* < 1 × 10^−5^. Each population was imputed separately with the TOPMed r2 panel^63–65^. After imputation, we removed first-degree relatives using KING, and variants with *R*^2^ < 0.8, MAF < 0.01, and HWE *P* < 1 × 10^−5^. Principal components were calculated with FlashPCA^70^. For cohorts with large case/control imbalances (Admixed American and US-African), we used the SAIGE logistic mixed model^69^ for calculating *p* value and generating summary statistics. Association tests for European, South, and East Asian were completed using PLINK v1.9 under an additive model with principal component adjustment to account for population stratification^62^.

#### GWAS data from Kobe University: Japanese, Korean, and Thai

Quality control and analysis of the Japanese dataset are previously described in Jia et al.^23^. Samples were filtered for call rate <97%, ambiguous sex, and IBD > 0.1875. Variants were filtered for info score >0.5, missing >3%, MAF > 0.5%, and HWE *P* ≥ 0.0001 in controls. SNPs were imputed with a Japanese reference panel with IMPUTE4 (v2.3.1). For the Thai dataset, SNPs with MAF < 0.005, call rate <97%, or HWE *P* < 1 × 10^−5^ were removed. Individuals with missing rate > 3%, IBD > 0.1875 and PCA outliers were removed. For the Korean dataset, SNPs with MAF < 0.01, call rate <99%, or HWE *P* < 5×10^−8^ for cases and <1 × 10^−5^ in controls were removed. Individuals with missing rate >4% or IBD (PI_HAT) > 0.2. No outliers were removed from PCA inspection. Both Thai and Korean genotypes were imputed with the 1000 Genomes reference panel using SHAPEIT^71^ and IMPUTE2^72^ and SNPs were filtered for info score <0.9 and 0.8 in Thai and Korean, respectively. Logistic regression was performed with Plink v1.9. Sex and the first four principal components were used in the Japanese cohort. No covariates were adjusted for the Thai and Korean datasets and *p* values were adjusted for genomic control (GC).

### Population-specific and multi-population meta-analysis

For each population-specific meta-analysis and the multi-population meta-analysis, we conducted an inverse-variance, fixed-effect meta-analysis using METAL (v2011-03-25) with adjustment for population stratification (GC) on each input dataset and assessment for heterogeneity selected^73^. For within-population meta-analyses, we removed variants with heterogeneity *p* value <0.05. All significant associations were visually inspected and single SNPs that did not follow the expected LD trend and SNPs with within-population heterogeneity removed.

For the European meta-analysis, we included summary statistics of only suggestive SNPs from a published GWAS in which the full data was not available^24^, increasing the European sample size to 1096 cases and 12,459 controls.

### Multi-population meta-regression with MR-MEGA

To account for and assess heterogeneous loci, we conducted a meta-regression using MR-MEGA v0.2^28^. We included three principal components, which captured the population structure across all twelve datasets. This allowed us to stratify heterogeneity into residual heterogeneity and heterogeneity that correlates with population. For each variant with heterogeneity that correlated with population, we visualized the dataset PCs from MR-MEGA with the dataset-specific log odds ratio from METAL. We adjusted for genomic control at the study level and after meta-regression to account for population structure within and between datasets. SNPs present in less than five studies were excluded. GC lambda (GC_λ_) was used to assess inflation. Results tables include summaries from both METAL and MR-MEGA analyses (Table 1). All Manhattan plots were generated with the qqman R package v0.4.1 [doi: 10.21105/joss.00731.] and LocusZoom web tool^74^. All significant loci are >1 Mb from each other with *r*^2^ < 0.1. Loci are labeled by nearest genes.

### Conditional analyses

To identify independent secondary significant loci at the candidate loci, we used GCTA COJO (v.1.93.2beta)^75,76^ to conduct approximate conditional analyses based on cohort-specific meta-analysis summary statistics. Conditional analysis was conducted in each dataset, with an LD reference generated from the dataset samples, due to differences in linkage disequilibrium structure between continental populations. Each cohort was conditioned for the eight independent loci identified from the initial meta-analysis. Multi-population meta-analysis of the conditioned cohorts was repeated in METAL^73^ to assess multi-population genome-wide significant secondary loci after GCTA.

### Heritability estimates

SNP-based heritability was estimated on a liability scale with LD score regression (LDSC v1.0.1)^77^ using a population prevalence of 16/100,000 and excluding HLA [chr6:25,000,000-34,000,000]. We used non-GC corrected population-specific meta-analysis summary statistics from METAL and pre-computed LD scores generated from the 1000 Genomes EUR or EAS samples. (https://alkesgroup.broadinstitute.org/LDSCORE/).

### Colocalization of SSNS GWAS variants and eQTLs datasets

We used fast enrichment estimation aided colocalization analysis (fastENLOC v1)^78^ for colocalization analysis with glomerular (*n* = 240) and tubulointerstitial (*n* = 311) eQTLs from nephrotic syndrome patients^79^, GTEx tissues (varied sample sizes), and immune eQTLs from both BLUEPRINT^46^ (*n* = 200) and DICE^45^ (*n* = 91) databases. Posterior probabilities for SSNS GWAS variants were calculated from MR-MEGA Z-scores using TORUS^80^. We used an LD panel from European and East Asian 1000 Genomes samples to define haplotype blocks in the pSSNS meta-analysis^60,81^. Enrichment of pSSNS GWAS variants in each eQTL dataset was estimated using fastENLOC and subsequently informed prior probabilities for each analysis. For colocalization with our kidney eQTLs, which had available raw data, we could identify multiple eQTLs per gene and multiple colocalized eQTLs at each locus. For all other data, in which only summary statistics were available, we assumed at most one colocalized SNP per loci.

### Open chromatin annotation of credible sets

95% credible sets were constructed for each independent locus identified from the multi-population meta-regression with Bayes’ factors reported by MR-MEGA. Posterior inclusion probability (PIP) was estimated by dividing each Bayes’ factor by the summation of Bayes’ factors across all variants within 1 Mb from the lead locus^82^.

SNPs within 95% credible sets of our genome-wide significant loci were evaluated for positional overlap based on the boundaries of known open chromatin peaks in kidney^50^ and immune^49^ cell types. For immune open chromatin, 76 samples from primary whole blood were used resulting in 1000–100,000 FACS-purified cells (GSE74912). For kidney, kidney cortex from 5 patients undergoing nephrectomies, resulting in 35,286 cells (GSE151302). Open chromatin peaks were identified by the MACS2 (v2.2.7.1) peak calling algorithm and optimized by gkmQC (v1.0)^51^.

### HLA imputation and analysis

To fine-map the HLA region, we conducted HLA imputation with the four-digit multi-ethnic v2 reference panel on Michigan Imputation Server^52^. Cohorts were imputed individually to optimize population-specific structure within the HLA region. The imputed cohorts were then merged for multi-population associations. We used HLA-TAPAS (v2020.05.02) ‘assoc’ module to conduct a logistic regression of the HLA region of the multi-population and population-specific datasets. For population-specific analyses, we adjusted for genotype-based principal components from Plink v1.9^62^. The population-specific principal components and continental populations were included as covariates in the multi-population analysis. HLA-TAPAS was also used to conduct a stepwise conditional analysis, conditioning on the locus with the smallest association *p* value. We additionally performed an omnibus test on the population-specific and multi-population cohorts to assess significance by amino-acid position.

### HLA modeling

To predict the reference (with arginine at position 47 and serine at position 52) structure of *HLA-DQA1* we extracted the sequence of *HLA-DQA1* from UNIPROT database (Uniprot ID: P01909). We used NCBI BLAST against PDB database to find the closest structure associated with the amino-acid sequence of P01909. We identified the top hit as 6PX6_A (HLA-TCR complex, *E* = 2x10^−161^) for the *HLA-DQA1* sequence^83^. We extracted the PDB coordinates for chain A from the 6PX6 and visualized in PYMOL v2.5. Since the most common amino-acid haplotype in the control population was arginine (47) and serine (52), we performed mutagenesis using PYMOL to model the reference protein 3D structure^84^.

In brief, we used the mutagenesis tool from PYMOL and selected the rotamer (most likely amino-acid conformation) for arginine and serine which showed the minimum number of clashes with nearby atoms. Afterwards, we adjusted the conformation of nearby atoms (within 5 Angstrom) to minimum free state using ‘Clean’ command in PyMOL which uses MMFF94 force field^85^. Though point mutations locally affect the conformation of the protein, they can result in torsion, bending and stretching of the entire molecule. Therefore, we exported the protein structure to SPDBV software for further refinement^86^.

We first fixed all the side chains of all amino acids to the best rotamer conformation using the simulated annealing method. Afterwards, we performed energy minimization using GROMOS 96 force field to extract the 3D coordinates that represent the lowest minimum energy conformation^87^. The refined protein structure of HLA-DQA1 was then assessed for changes in stability of protein for both amino-acid combinations for each haplotype using “MULTIPLE MUTATION” in DynMut2 server^55^. The instability of HLA-DQA1 was evaluated using the predicted ddG parameter which measures changes in Gibbs free energy between the folded and unfolded states and the change in folding when a mutation is present. The interaction among amino acids in reference and mutated structure were predicted using Arpeggio^88^ and visualized in PyMOL.

### Polygenic risk score analysis

#### Construction of the multi-population PRS

To investigate genetic risk across the genome, we generated a PRS using 1974 cases and 20,039 controls from the GWAS of European (US-European), East Asian (Japanese, Korean, US-East Asian), African (US-AFR, EU-AFR), and South Asian (US-SAS, Indian) populations using PRS-CSx (v7-29-2021)^89^. Populations with less than 100 cases (Maghrebian, Admixed American) were excluded. The EU-EUR dataset was excluded from PRS calculations and was used as an independent test/train dataset, where 80% of cases and controls were randomly selected for training and 20% for testing. While our goal of this analysis was to explore the relationships between the PRS and clinical correlates within case cohorts, not case/control prediction, we used prediction accuracy to optimize the gamma-gamma priors and the global shrinkage parameter used in the PRS-CSx model. We varied the hyper parameters and chose the model with the best prediction accuracy (F-measure; Supp. Table 15). The regression betas from the best model were used to weight the population-specific PRS.

#### Construction of the European PRS

We also generated a PRS from the GWAS of European (US-European) populations using PRS-CS^90^. Similar to the multi-population method, the EU-EUR dataset was used as an independent test/train dataset, where 80% of cases and controls were randomly selected for training and 20% for testing. We used prediction accuracy to optimize the gamma-gamma priors and the global shrinkage parameter used in the PRS-CS model. We varied the hyperparameters and chose the model with the best prediction accuracy (F-measure; Supp. Table 14).

#### Clinical associations with PRS

The PRS was applied to pediatric participants from the EU-European data for which clinical data were available (*n* = 233). For each PRS (European and multi-population), we split the samples into PRS quantiles. We tested significance of PRS quartiles in the following models: Model 1: sex ~ PRS + age of onset + relapse pattern + 4PCs (multiple logistic regression); Model 2: relapse pattern ~ PRS + age of onset + sex + 4PCs (multiple logistic regression); Model 3: age of onset ~ PRS + sex + relapse pattern + 4PCs (multiple linear regression). The effect size, standard error, and *p* value for the PRS effect are reported.

### Reporting summary

Further information on research design is available in the Nature Portfolio Reporting Summary linked to this article.

## Supplementary information

Supplementary Information

Reporting Summary

## Data Availability

The fixed-effects multi-population summary statistics (METAL) generated by this study have been deposited in the GWAS Catalog [GCST90258619]. The credible sets for significant variants and significant colocalization results generated in this study are provided in the Supplementary file. The raw GWAS data are protected and are not available due to data privacy laws. For HLA modeling, we used the Protein Data Bank (PDB; https://www.rcsb.org/) and UNIPROT database (Uniprot ID: P01909). For colocalization analyses, we used the NEPTUNE cohort, the unfiltered eQTL results from DICE, and the BLUEPRINT consortium.

## Code availability

Publicly available software was used to perform the analyses. We will be happy to share some or all our code with any requester.
