# Supplementary material for: Multi-population genome-wide association study implicates immune and non-immune factors in pediatric steroid-sensitive nephrotic syndrome
Source: Nat Commun. 2023 Apr 29;14:2481. doi: 10.1038/s41467-023-37985-w (PMC10148875; doi:10.1038/s41467-023-37985-w)
Supplement: Supplementary file 1 — Supplementary Information [file 41467_2023_37985_MOESM1_ESM.pdf]

SUPPLEMENTAL MATERIAL

Multi-population genome-wide association study implicates both immune and non-immune factors in the etiology of pediatric steroid sensitive nephrotic syndrome

Alexandra Barry[1,2+], Michelle T. McNulty[1,2+], Xiaoyuan Jia[3,4+], Yask Gupta[5+], Hanna Debiec[6+], Yang Luo[7,8,9,10], China Nagano[1,2,11], Tomoko Horinouchi[11], Seulgi Jung[12], Manuela Colucci[13], Dina F. Ahram[5], Adele Mitrotti[5,14], Aditi Sinha[15], Nynke Teeninga[16], Gina Jin[5], Shirlee Shril[17,18], Gianluca Caridi[19], Monica Bodria[20], Tze Y Lim[5], Rik Westland[21], Francesca Zanoni[5,22], Maddalena Marasa[5], Daniel Turudic[23], Mario Giordano[24], Loreto Gesualdo[14], Riccardo Magistroni[25,26], Isabella Pisani[27], Enrico Fiaccadori[27], Jana Reiterova[28], Silvio Maringhini[29], William Morello[30], Giovanni Montini[30,31], Patricia L. Weng[32], Francesco Scolari[33], Marijan Saraga[34], Velibor Tasic[35], Domenica Santoro[36], Joanna A.E. van Wijk[21], Danko Milošević[37, 23], Yosuke Kawai[3,4], Krzysztof Kiryluk[5], Martin R. Pollak[38,39], Ali Gharavi[5], Fangmin Lin[39], Ana Cristina Simœs e Silva[40], Ruth J.F. Loos[41], Eimear E. Kenny[42,43,44], Michiel F. Schreuder[16], Aleksandra Zurowska[45], Claire Dossier[46], Gema Ariceta[47], Magdalena Drozynska-Duklas[45], Julien Hogan[46], Augustina Jankauskiene[48], Friedhelm Hildebrandt[1,18], Larisa Prikhodina[49], Kyuyoung Song[12], Arvind Bagga[15], Hae II Cheong[50], Gian Marco Ghiggeri[20] , Prayong Vachvanichsanong[51], Kandai Nozu[11], Dongwon Lee[1,2,18], Marina Vivarelli[52], Soumya Raychaudhuri[8,9,10,53,54], Katsushi Tokunaga[3,4*], Simone Sanna-Cherchi[5*], Pierre Ronco[6,55*], Kazumoto Iijima[56,57*], Matthew G. Sampson[1,2,18, 58*]

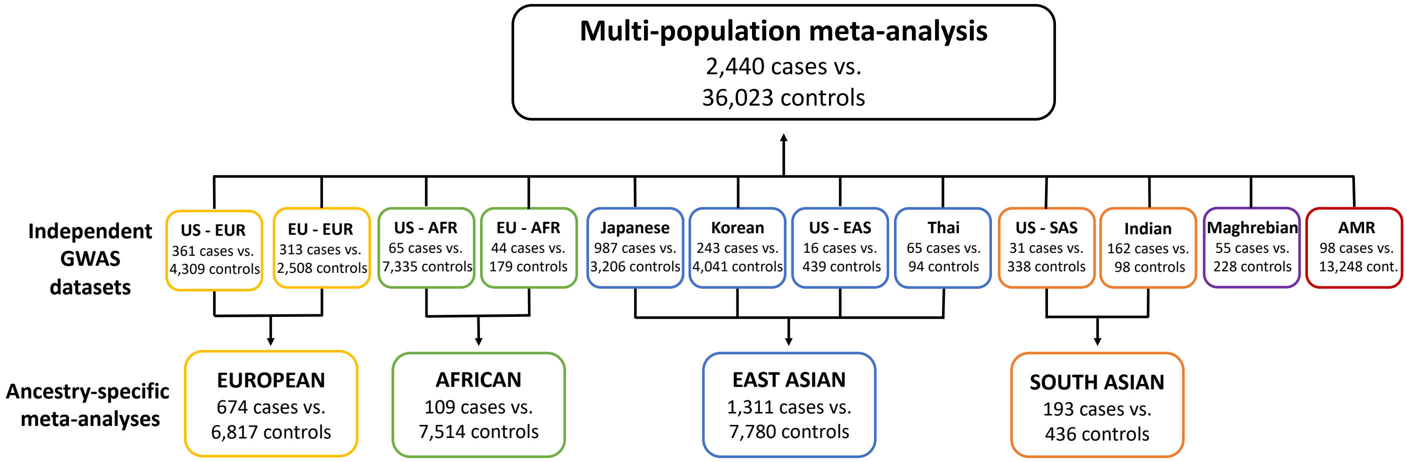

Supplementary Figure 1. Flow-chart of GWAS datasets and analyses
Individual datasets were used as input for the multi-population meta-analysis. EUR = European, AFR = African, EAS = East Asian, SAS = South Asian, AMR = Admixed American.

Supplementary Table 1. Summary of GWAS datasets

				Pre-QC		Post-QC							
GWAS Cohort	Population	Source	Genotype Platform	n cases	n controls	n cases	n controls	n total	Imputation Reference panel	n SNPs	GWAS model	GC-γ	Regression adjustments
Nephrovir/ EU	European	New data + 244 cases and 2,552 controls from Debiec et al.	Cases: Illumina Omni 2.5, Illumina Human OmniExpress & InfiniumOmni2-5-8v1-3<br><br>Controls: Illumina Human OmniExpress (3Cites) & Omni 2.5 (1000 Genomes)	626	2,775	313	2,508	2,821	TOPMed r2	8,112,877	Plink v1.9 / Logistic regression	1.05	3 PCs
US Cohort	European	New	Cases: MEGA<br><br>Controls: MEGA1.0 (PAGE)	371	4,359	361	4,309	4,670	TOPMed r2	8,316,416	Plink v1.9 / Logistic regression	1.06	5 PCs
US Cohort	African	New	Cases: MEGA<br><br>Controls: MEGA1.0 (PAGE)	65	7,344	65	7,335	7,400	TOPMed r2	13,421,506	SAIGE / Logistic mixed model	0.84	2 PCs
Nephrovir/ EU	African	Reanalyzed from Debiec et al.	Cases: Illumina Human OmniExpress<br><br>Controls: Omni 2.5 (1000 Genomes)	56	451	44	179	223	TOPMed r2	12,413,167	Plink v1.9 / Logistic regression	1.02	5 PCs
US Cohort	East Asian	New	Cases: MEGA<br><br>Controls: MEGA1.0 (PAGE)	17	443	16	439	455	TOPMed r2	6,196,585	Plink v1.9 / Logistic regression	1.00	4 PCs
Japanese	East Asian	Summary statistics from Jia et al.	Cases and controls: Affymetrix Japonica array	1,01 8	3,331	987	3,206	4,193	2KJPN panel w/ IMPUTE4	6,088,373	Plink v1.9 / Logistic regression	1.05	Sex, 4 PCs
Korean	East Asian	New	Cases:Axiom Array<br><br>Controls: Illumina OmniQuad chip	249	4,041	243	4,041	4,284	1000 Genomes reference panel w/ SHAPEIT and IMPUTE2	2,912,065	Plink v1.9 / Logistic regression	1.03	None (used GC-adj P-values)
Thai	East Asian	New	Cases and controls: Axiom array	66	96	65	94	159	1000 Genomes reference panel w/ SHAPEIT and IMPUTE2	4,946,221	Plink v1.9 / Logistic regression	1.00	None (used GC-adj P-values)
US Cohort	South Asian	New	Cases: MEGA<br><br>Controls: MEGA1.0 (PAGE)	39	534	31	338	369	TOPMed r2	7,637,355	Plink v1.9 / Logistic regression	0.93	3 PCs
Indian	South Asian	New	Cases and controls: Infinium Omni2.5	170	109	162	98	260	TOPMed r2	8,159,073	Plink v1.9 / Logistic regression	1.03	3 PCs
Maghrebian	Maghrebian	Reanalyzed from Debiec et al.	Cases and controls: Illumina Human OmniExpress	85	261	55	228	283	TOPMed r2	10,072,905	Plink v1.9 / Logistic regression	0.90	3 PCs
Admixed American	Admixed American	New	Cases: MEGA<br><br>Controls: MEGA1.0 (PAGE)	109	13,266	98	13,248	13,346	TOPMed r2	9,264,840	SAIGE / Logistic mixed model	0.98	3 PCs
Total						2,440	36,023	38,463					

This table summarizes samples, quality control and methods for individual GWAS datasets. Sample quality control (QC) varies by study, see Methods. GC-γ = Genomic control lambda, PC = genetic principal components.

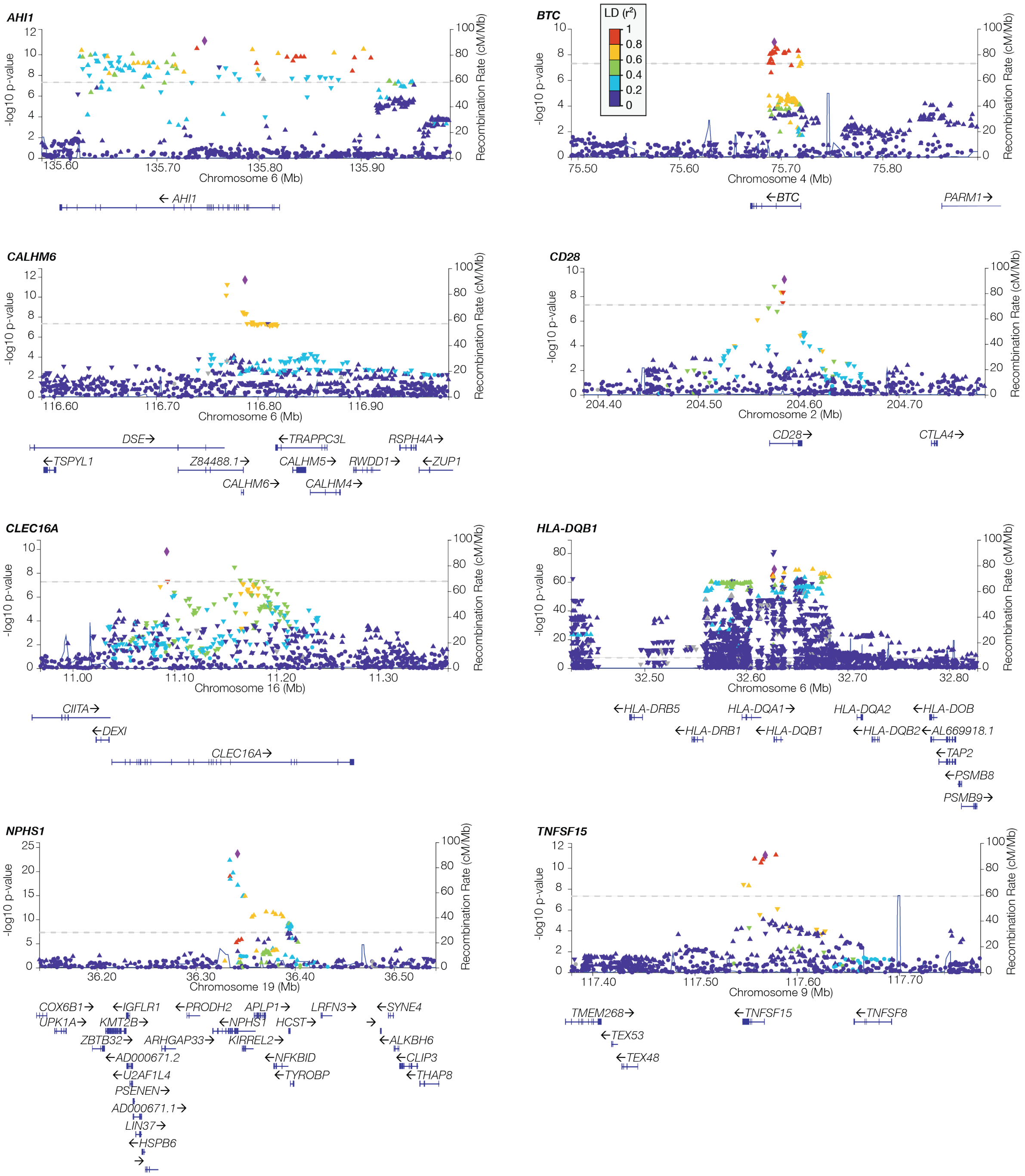

Supplementary Figure 2. LocusZoom plots of genome-wide significant loci

Each figure is titles by the closest gene to the significant locus. Linkage disequilibrium (LD/r^2) with the most significant SNP in the reference panel (purple diamond) is estimated from all 1000 Genomes populations. *P*-values are from a test of deviance of the full meta-regression model compared to the null model using MR-MEGA (two-sided). The dashed horizontal line indicates the genome-wide significance threshold ($5x10^{-8}$).

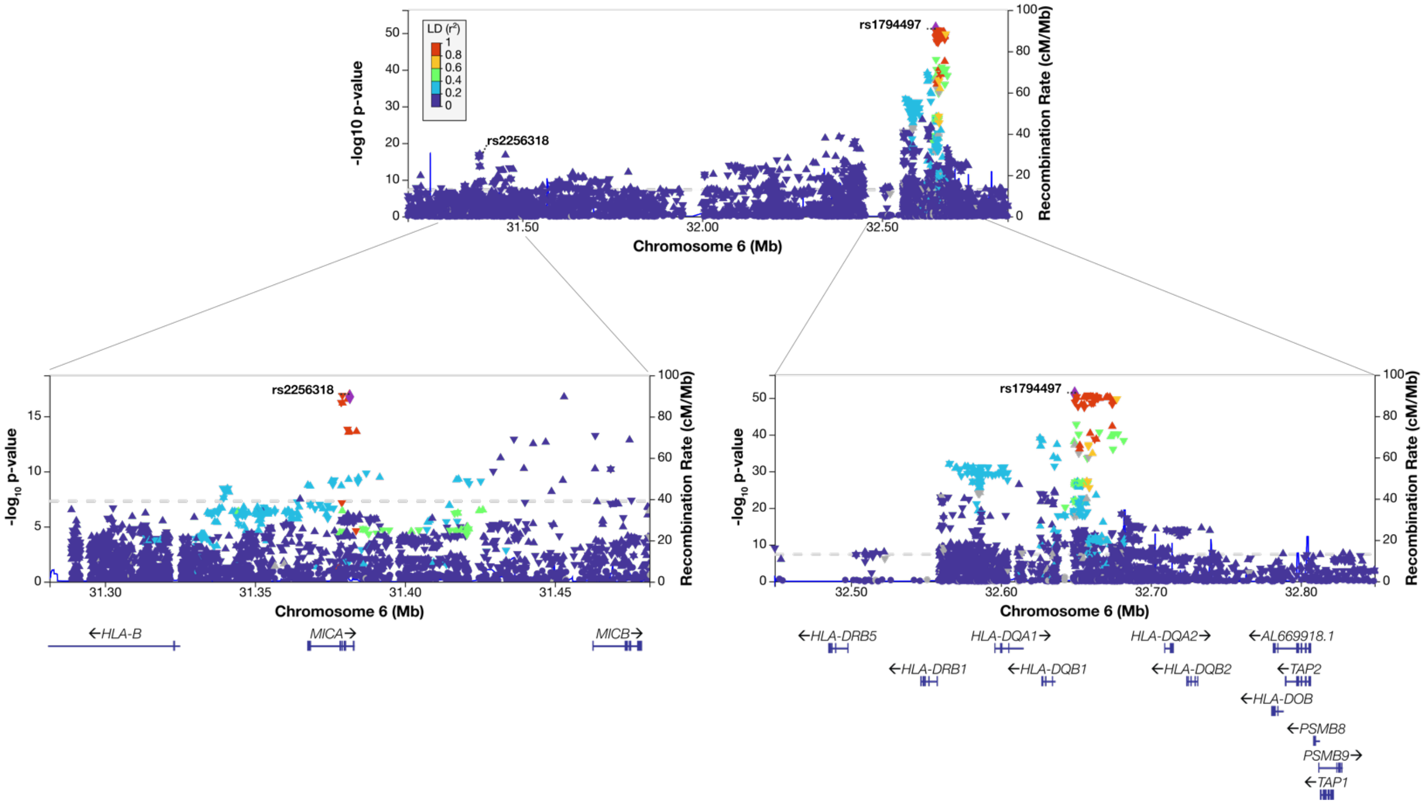

Supplementary Figure 3. LocusZoom plot of significant loci from conditional analysis

The p-values are from multiple linear regression with COJO (two-sided). Analysis conditioned on: rs55730955, rs28862935, rs1063355, rs2637681, rs7759971, rs10817678, rs8062322, and rs56117924. rs2256318 and rs1794497 are ~1.3Mb apart with $r^2 < 0.13$ across all the 1000 Genomes Project populations, and with an $r^2 = 0.04$ when combining all the 1000 Genomes Project samples.

Supplementary Table 2. Summary of population-specific meta-analyses

GWAS Cohort	n cases	n controls	n total	GC-γ	n SNPs
African	109	7,514	7,623	0.85	14,302,064
East Asian	1,311	7,780	9,091	0.99	8,644,038
European	674	6,817	7,491	1.07	8,414,111
South Asian	193	436	629	0.95	8,460,574

GC-γ genomic control lambda. Admixed American and Maghrebian excluded from population-specific meta-analyses since they only have one cohort.

Supplementary Table 3. Genome-wide significant SNPs from population-specific meta-analyses

Population	Top SNP	Position (hg19)	Nearest Gene	EA	NEA	OR [95% CI]	P
East Asian	rs9274740	6:32637968	*HLA-DQB1*	A	T	0.41 [0.36, 0.47]	1.25×10^{-39}
East Asian	rs412175	19:36342103	*NPHS1*	T	C	0.53 [0.47, 0.60]	7.47×10^{-25}
East Asian	rs2596485	6:31364870	*MICA*	T	C	0.61 [0.53, 0.69]	2.46×10^{-15}
East Asian	rs7848647	9:117569046	*TNFSF15*	T	C	0.70 [0.63, 0.77]	9.57×10^{-13}
East Asian	rs1181388	2:204575951	*CD28*	A	G	0.73 [0.66, 0.80]	4.93×10^{-11}
East Asian	rs115180879[a]	6:28984623	*ZNF311*	T	C	3.00 [2.09, 4.32]	3.10×10^{-9}
East Asian	rs8062322	16:11092319	*CLEC16A*	A	C	0.68 [0.59, 0.77]	5.48×10^{-9}
European	rs9271747	6:32626037	*HLA-DQB1*	T	G	3.30 [2.82, 3.86]	5.69×10^{-51}
European	rs2637678[b]	6:116787378	*CALHM6*	T	C	1.74 [1.58, 1.93]	2.35×10^{-27}
European	rs2857607[a]	6:31517248	*NFKBIL1*	T	C	1.95 [1.66, 2.31]	1.18×10^{-13}
European	rs2746419[b]	6:135653855	*AHI1*	A	C	1.34 [1.22, 1.47]	1.63×10^{-10}
European	rs111796602[b]	7:37402490	*ELMO1**	T	C	0.68 [0.59, 0.78]	1.72×10^{-8}
European	rs12911841	15:79162355	*MORF4L1**	T	C	2.95 [2.01, 4.34]	3.88×10^{-8}

[a] SNPs are > 1Mb from HLA; however, because this region is known for long-range LD, they should be interpreted with caution. These SNPs are not included in our total count of discovered loci.
[b] European meta-analysis includes the limited summary statistics available from the GWAS Catalogue from Dufek et.al., for a total of 1,096 cases and 12,459 controls.

The two-sided, fixed-effect inverse variance weighted meta-analyses were conducted with METAL. *P*-values are unadjusted for multiple testing. All SNP are > 1Mb from each other with $r^2 < 0.1$. Variants showing high within-population heterogeneity are excluded (HetPVal < 0.05). We found no genome-wide significant associations in the South Asian, African, Admixed American and Maghrebian meta-analyses. Novel loci are indicated with *.

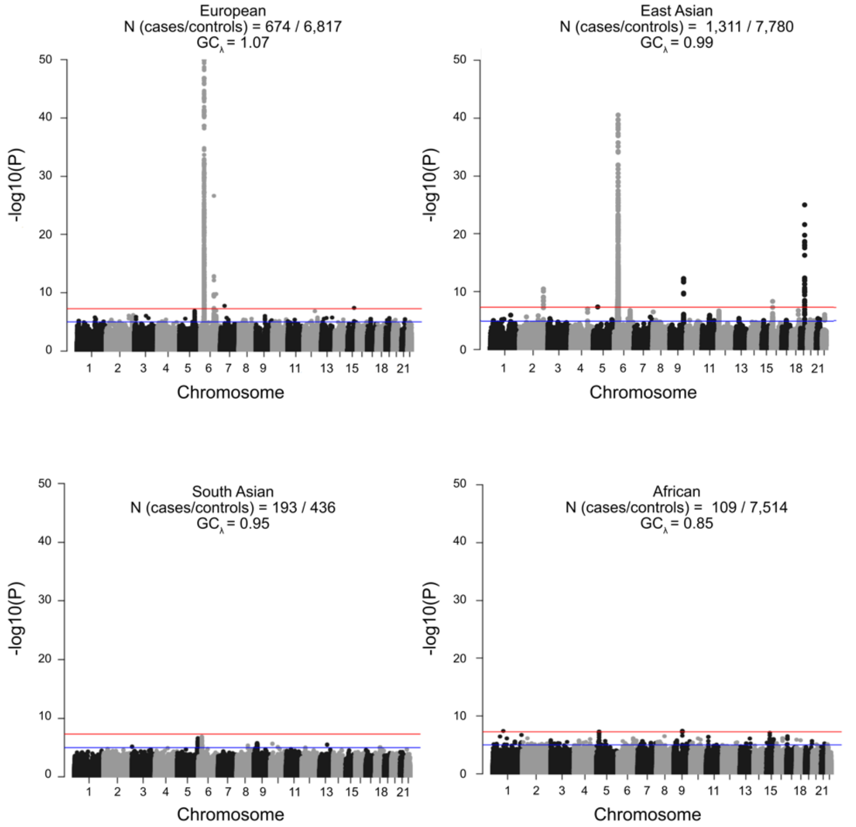

Supplementary Figure 4. Manhattan plots of ancestry-specific meta-analyses

The two-sided, fixed-effect inverse variance weighted meta-analyses were conducted with METAL. *P*-values are unadjusted for multiple testing. The red line indicates the genome-wide significance threshold (5 x 10^{-8}); the blue line is suggestive significance (1 x 10^{-5}). The number of cases and controls and genomic control lambda (GC_{γ}) are reported for each dataset.

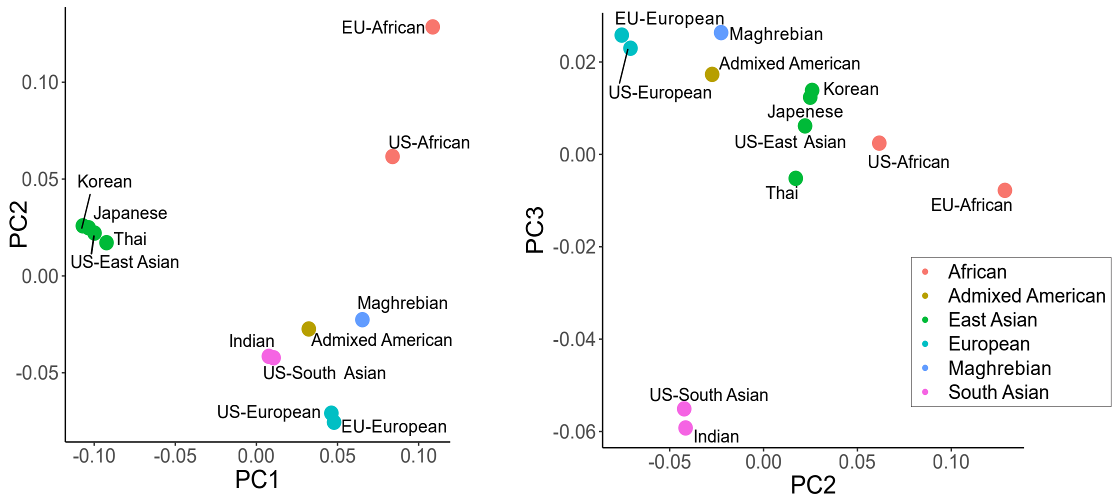

Supplementary Figure 5. Dataset principal components (PCs) used in MR-MEGA meta-regression analysis The dots are labeled by study and colored by population. PCs were generated from allele frequencies across all GWAS variants.

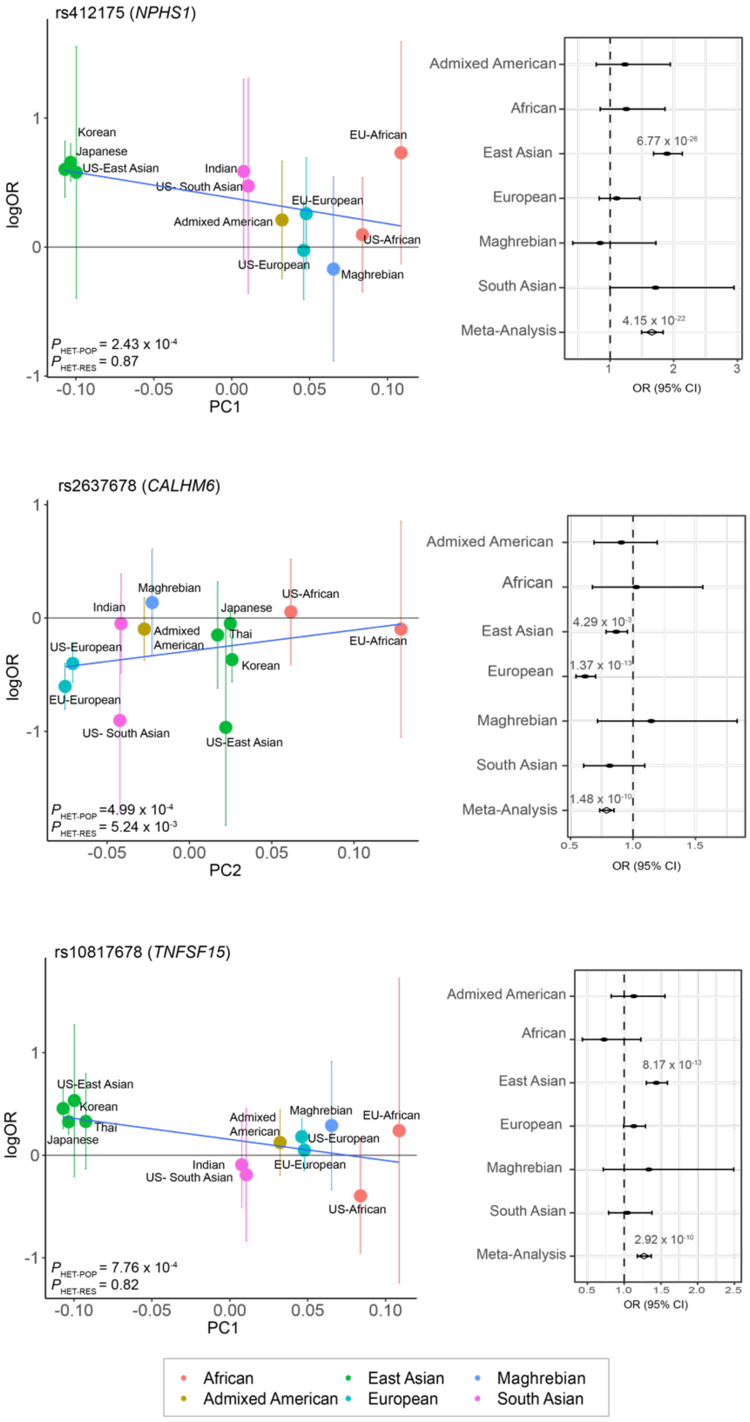

Supplementary Figure 6. Population-specificity of heterogeneous GWAS variants

Left: Correlation of MR-MEGA principal components (PCs) and logarithm of the odds ratio (logOR) with 95% confidence interval for each dataset GWAS. The nearest gene for each SNP is in parentheses. $P_{\text{HET-POP}}$ measures the heterogeneity in allelic effects that is correlated with population PCs and is from a test of deviance of the full model compared to the model excluding population principal components. The residual heterogeneity, $P_{\text{HET-RES}}$, is the deviance of the full model. **Right**: Multi-population and single-population odds ratios with 95% confidence interval for each SNP. Maghrebian and Admixed American *P*-values are from logistic regression, and the remainder are from inverse-variance fixed-effects meta-analysis with METAL. All P-values are two sided and unadjusted for multiple testing. Number of cases in each analysis : Admixed American: n = 98, African: n = 109, East Asian: n = 1,311, European: n = 674, Maghrebian: n = 55, South Asian: n = 193, Meta-Analysis: n = 2,440.

Supplementary Table 4. Suggestive SNPs (P-value$_{\text{MR-MEGA}}$ < 1 x 10^{-5}) from multi-population meta-analysis

					Fixed-effects meta-analysis			Meta-regression (MR-MEGA)		
Nearest Gene	Top SNP	Position (hg19)	EA	NEA	OR [95% CI]	P-value	Het χ^2 P-value	P-value	Ancestry Het P-value	Residual Het P-value
GSDMB	rs9303279	17:38073968	G	C	1.22 [1.13, 1.31]	1.88 x 10^{-7}	0.16	2.49 x 10^{-7}	0.05	0.43
TNFSF4	rs1012507	1:173219471	T	G	1.20 [1.11, 1.29]	5.73 x 10^{-6}	0.05	2.92 x 10^{-7}	2.52 x 10^{-3}	0.70
PHC1	rs1805732	12:9090892	T	C	0.82 [0.76, 0.88]	1.71 x 10^{-7}	0.51	4.65 x 10^{-7}	0.10	0.85
MORF4L1	rs12911841	15:79162355	T	C	1.82 [1.43, 2.30]	6.92 x 10^{-7}	0.03	5.82 x 10^{-7}	0.03	0.12
COL21A1	rs4715598	6:56105038	G	C	1.22 [1.10, 1.36]	3.37 x 10^{-4}	3.53 x 10^{-3}	9.31 x 10^{-7}	1.90 x 10^{-4}	0.49
CLIC4	rs4649032	1:25197586	T	C	1.20 [1.12, 1.30]	1.18 x 10^{-6}	0.16	1.18 x 10^{-6}	0.03	0.59
CSMD1	rs2469358	8:3451111	G	A	1.14 [1.05, 1.22]	5.56 x 10^{-4}	1.32 x 10^{-3}	1.38 x 10^{-6}	1.52 x 10^{-4}	0.18
OPN5	rs62397901	6:47746146	G	T	1.50 [1.27, 1.78]	2.34 x 10^{-6}	0.15	1.61 x 10^{-6}	0.02	0.91
KC6	rs16975006	18:39160883	T	G	1.68 [1.39, 2.02]	6.21 x 10^{-8}	0.39	1.64 x 10^{-6}	0.38	0.35
C16orf95	rs7197567	16:86980044	T	C	1.31 [1.18, 1.45]	3.85 x 10^{-7}	0.75	2.69 x 10^{-6}	0.23	0.90
COCH	rs12431424	14:31318179	A	T	0.78 [0.70, 0.87]	3.67 x 10^{-6}	0.15	3.14 x 10^{-6}	0.03	0.55
ABCB9	rs6489247	12:123385443	T	C	1.24 [1.12, 1.38]	7.47 x 10^{-5}	0.02	3.22 x 10^{-6}	1.99 x 10^{-3}	0.43
BAD	rs477895	11:64048912	C	T	1.19 [1.10, 1.29]	3.44 x 10^{-5}	0.07	3.81 x 10^{-6}	4.56 x 10^{-3}	0.75
SLC22A5	rs13190001	5:131744482	T	C	0.87 [0.80, 0.95]	1.31 x 10^{-3}	8.14 x 10^{-3}	4.08 x 10^{-6}	2.10 x 10^{-4}	0.71
IL15	rs6817696	4:142331333	C	A	1.17 [1.07, 1.28]	7.87 x 10^{-4}	0.01	4.09 x 10^{-6}	3.71 x 10^{-4}	0.51
RRP12	rs6584128	10:99171926	G	T	1.21 [1.13, 1.30]	1.74 x 10^{-7}	0.19	5.10 x 10^{-6}	0.75	0.08
NFIA	rs75447844	1:61807924	T	C	1.71 [1.30, 2.24]	1.12 x 10^{-4}	4.69 x 10^{-3}	7.36 x 10^{-6}	2.58 x 10^{-3}	0.17
CCDC66	rs190705792	3:56612599	T	G	2.47 [1.74, 3.49]	3.42 x 10^{-7}	0.34	7.62 x 10^{-6}	0.51	0.17
CD164	rs76615866	6:109618364	C	G	2.08 [1.56, 2.78]	6.69 x 10^{-7}	0.40	8.18 x 10^{-6}	0.30	0.44
SUGCT	rs75048637	7:41285435	T	C	1.52 [1.25, 1.85]	2.60 x 10^{-5}	0.03	8.33 x 10^{-6}	0.01	0.29

The fixed-effect meta-analysis was performed with METAL. Its P-value is from a two-sided, inverse variance weighted meta-analysis and the Het χ^2 P-value is from a two-sided, chi-square test for heterogeneity. The meta-regression was performed with MR-MEGA, and all tests are two-sided and approximated by a chi-square distribution. The MR-MEGA P-value tests is from test of deviance of the full meta-regression model compared to the null model. Its population Het P-value measures the heterogeneity in allelic effects that is correlated with GWAS populations and is from a test of deviance of the full model compared to the model excluding population principal components. The residual heterogeneity is the deviance of the full model. Conditional analysis was performed on all loci from the discovery meta-analysis, except for rs412175 and rs2637678. rs56117924 and rs2637681 were used in conditional analysis, respectively. rs2256318 and rs1794497 are ~1.3Mb apart with r^2< 0.13 across all the 1000 Genomes Project populations, and with an r^2 = 0.04 when combining all the 1000 Genomes samples. MR-MEGA results are not available for the conditional analysis. Novel loci *, EA effect allele, NEA non-effect allele, OR [95% CI]= Odds ratio with 95% confidence interval.

Supplementary Table 5. Allele frequency of genome-wide and suggestive significant SNPs across ancestries

Nearest Gene	Top SNP	EA	European Case n=674	European Control n=6,817	South Asian Case n=193	South Asian Control n=436	East Asian Case n=1,311	East Asian Control n=7,780	African Case n=109	African Control n=7,514	Maghrebian Case n=55	Maghrebian Control n=228	Admixed American Case n=98	Admixed American Control n=13,248
Genome-wide significant														
HLA-DQB1	rs1063355	T	0.22	**0.40**	0.35	**0.49**	0.28	**0.44**	0.28	**0.46**	0.15	**0.38**	0.17	**0.34**
NPHS1	rs412175	C	**0.05**	0.04	**0.10**	0.06	**0.24**	0.15	**0.46**	0.34	0.12	**0.14**	**0.18**	0.15
CALHM6	rs2637678	C	0.33	**0.43**	0.21	**0.26**	0.40	**0.44**	0.22	**0.29**	**0.52**	0.50	**0.43**	0.38
AHI1	rs7759971	T	**0.40**	0.35	**0.42**	0.39	**0.26**	0.22	0.19	**0.21**	**0.35**	0.21	**0.37**	0.30
TNFSF15	rs10817678	G	0.29	**0.33**	**0.26**	0.22	0.34	**0.44**	0.13	**0.17**	0.15	**0.18**	0.23	**0.24**
CLEC16A	rs8062322	A	0.31	**0.34**	0.30	**0.38**	0.14	**0.19**	**0.46**	0.44	0.20	**0.34**	**0.33**	0.30
CD28	rs55730955	A	0.05	**0.06**	0.09	0.09	0.45	**0.55**	0.07	**0.08**	0.05	**0.10**	0.08	**0.11**
BTC	rs28862935	A	**0.23**	0.16	**0.28**	0.25	**0.06**	0.05	**0.48**	0.36	**0.44**	0.30	**0.34**	0.23
HLA-DQB1	rs1794497	C	**0.28**	0.12	**0.45**	0.30	**0.20**	0.16	**0.15**	0.11	**0.35**	0.16	**0.25**	0.11
MICA	rs2256318	A	**0.30**	0.22	**0.46**	0.27	**0.27**	0.23	**0.27**	0.23	**0.45**	0.38	**0.38**	0.25
Suggestive significant														
GSDMB	rs9303279	G	**0.50**	0.45	0.41	**0.42**	**0.30**	0.26	0.11	**0.21**	**0.42**	0.33	**0.35**	0.34
TNFSF4	rs1012507	T	**0.37**	0.34	0.32	0.32	**0.25**	0.23	0.24	**0.29**	0.21	**0.26**	0.34	**0.39**
PHC1	rs1805732	T	0.40	**0.43**	0.47	0.47	0.42	**0.48**	0.15	**0.21**	0.30	**0.38**	0.35	**0.38**
MORF4L1	rs12911841	T	**0.03**	0.01	-	-	-	-	**0.27**	0.18	0.18	0.18	**0.10**	0.05
COL21A1	rs4715598	G	**0.55**	0.50	**0.39**	0.35	**0.43**	0.24	**0.43**	0.40	**0.61**	0.53	**0.48**	0.42
CLIC4	rs4649032	T	**0.50**	0.49	**0.43**	0.35	**0.49**	0.44	**0.44**	0.40	0.47	**0.48**	**0.44**	0.41
CSMD1	rs2469358	G	0.31	**0.33**	0.34	**0.36**	**0.39**	0.36	**0.33**	0.27	0.22	0.22	0.23	**0.29**
OPN5	rs62397901	G	**0.09**	0.06	**0.14**	0.10	0.01	0.01	-	-	**0.07**	0.05	**0.12**	0.04
KC6	rs16975006	T	**0.03**	0.02	**0.04**	0.02	0.01	0.01	**0.25**	0.20	**0.12**	0.06	**0.12**	0.06
C16orf95	rs7197567	T	**0.09**	0.06	0.06	**0.07**	**0.21**	0.18	**0.54**	0.42	**0.23**	0.19	**0.22**	0.20
COCH	rs12431424	A	0.13	**0.15**	0.10	**0.15**	0.16	**0.18**	0.12	**0.19**	**0.12**	0.10	0.09	**0.21**
ABCB9	rs6489247	T	**0.12**	0.09	**0.27**	0.19	0.17	**0.18**	0.10	0.10	**0.10**	0.07	**0.09**	0.07
BAD	rs477895	C	**0.19**	0.18	0.26	**0.27**	**0.25**	0.21	**0.56**	0.43	**0.49**	0.34	**0.31**	0.21
SLC22A5	rs13190001	T	0.36	**0.44**	0.17	**0.21**	**0.26**	0.25	0.28	**0.32**	**0.33**	0.31	0.34	**0.45**
IL15	rs6817696	C	**0.37**	0.35	0.28	**0.30**	**0.08**	0.06	0.28	**0.33**	0.36	**0.40**	**0.33**	0.27
RRP12	rs6584128	G	**0.37**	0.34	**0.42**	0.40	**0.36**	0.30	**0.64**	0.53	**0.55**	0.51	**0.43**	0.37
NFIA	rs75447844	T	0.02	0.02	**0.09**	0.05	0.13	**0.15**	-	-	0.01	0.01	0.06	**0.14**
CCDC66	rs190705792	T	**0.03**	0.01	-	-	-	-	**0.06**	0.03	**0.04**	0.02	**0.04**	0.01
CD164	rs76615866	C	-	-	-	-	-	-	**0.27**	0.16	0.03	0.03	**0.07**	0.04
SUGCT	rs75048637	T	**0.06**	0.04	0.03	**0.04**	-	-	**0.16**	0.09	**0.22**	0.14	**0.09**	0.06

The case/control group with the higher frequency is indicated in bold. EA effect allele. Nearest gene is limited to protein coding genes. Genome-wide significant = $P_{\text{MR-MEGA}} < 5 \times 10^{-8}$. Exact *P*-values in Supplementary Data 4. Suggestive significant = $P_{\text{MR-MEGA}} < 1 \times 10^{-5}$. *P*-values for each SNP are in Table 1 and Supplementary Table 4.

Supplementary Table 6. SNPs that significantly colocalize with tissue and cell type eQTLs

RS ID	Position (hg19)	Ensembl Gene ID	Gene Symbol	Study	Tissue/Cell type	RCP	SCP
rs5024432	6:32684468	ENSG00000237541	*HLA-DQA1*	GTEx	Skin (not sun exposed)	0.22	0.13
rs2637678	6:116787378	ENSG00000188820	*CALHM6**	DICE	Monocytes, classical	0.86	0.86
rs2637681	6:116769845	ENSG00000188820	*CALHM6**	GTEx	Adipose visceral omentum	0.39	0.39
rs2637681	6:116769845	ENSG00000188820	*CALHM6**	GTEx	Skin (not sun exposed)	0.25	0.25
rs7759971	6:135746884	ENSG00000135541	*AHI1**	DICE	Monocytes, classical	0.65	0.23
rs7759971	6:135746884	ENSG00000135541	*AHI1**	BLUEPRINT	Monocyte	0.26	0.10
rs6908428	6:135793706	ENSG00000135541	*AHI1**	DICE	T cell, CD4, memory TREG	0.65	0.62
rs6908428	6:135793706	ENSG00000135541	*AHI1**	DICE	T cell, CD4, naive	0.62	0.62
rs6908428	6:135793706	ENSG00000135541	*AHI1**	DICE	T cell, CD4, naive TREG	0.57	0.57
rs6908428	6:135793706	ENSG00000135541	*AHI1**	DICE	T cell, CD4, TFH	0.56	0.56
rs6908428	6:135793706	ENSG00000135541	*AHI1**	DICE	B cell, naive	0.56	0.56
rs6908428	6:135793706	ENSG00000135541	*AHI1**	DICE	Monocytes, non-classical	0.53	0.52
rs6908428	6:135793706	ENSG00000135541	*AHI1**	DICE	T cell, CD4, TH17	0.51	0.51
rs6908428	6:135793706	ENSG00000135541	*AHI1**	DICE	T cell, CD8, naive	0.40	0.40
rs6908428	6:135793706	ENSG00000135541	*AHI1**	DICE	T cell, CD4, TH1/17	0.39	0.39
rs6908428	6:135793706	ENSG00000231028	*LINC00271*	DICE	T cell, CD4, memory TREG	0.36	0.07
rs6908428	6:135793706	ENSG00000234084	*Lnc-MYB-2*	DICE	T cell, CD4, naive	0.66	0.26
rs6908428	6:135793706	ENSG00000234084	*Lnc-MYB-2*	DICE	B cell, naive	0.52	0.51
rs7759971	6:135746884	ENSG00000234084	*Lnc-MYB-2*	DICE	Monocytes, non-classical	0.42	0.17
rs6908428	6:135793706	ENSG00000234084	*Lnc-MYB-2*	DICE	T cell, CD4, TH1/17	0.33	0.32
rs6908428	6:135793706	ENSG00000234084	*Lnc-MYB-2*	DICE	T cell, CD4, TFH	0.33	0.20
rs6908428	6:135793706	ENSG00000234084	*Lnc-MYB-2*	DICE	T cell, CD4, naive TREG	0.31	0.19
rs6908428	6:135793706	ENSG00000234084	*Lnc-MYB-2*	DICE	T cell, CD4, memory TREG	0.22	0.09
rs6908428	6:135793706	ENSG00000234084	*Lnc-MYB-2*	DICE	T cell, CD4, TH17	0.21	0.10
rs7848647	9:117569046	ENSG00000181634	*TNFSF15**	DICE	Monocytes, classical	0.95	0.28
rs10817678	9:117579457	ENSG00000181634	*TNFSF15**	BLUEPRINT	Monocyte	0.90	0.69
rs6478108	9:117558703	ENSG00000181634	*TNFSF15**	GTEx	Whole blood	0.62	0.43
rs6478109	9:117568766	ENSG00000181634	*TNFSF15**	GTEx	Artery - aorta	0.57	0.30
rs7848647	9:117569046	ENSG00000181634	*TNFSF15**	DICE	Monocytes, non-classical	0.22	0.05
rs12941333	17:38040534	ENSG00000172057	*ORMDL3*	DICE	T cell, CD4, memory TREG	0.23	0.02
rs8076131	17:38080912	ENSG00000073605	*GSDMB**	DICE	T cell, CD4, memory TREG	0.29	0.16

Top colocalized SSNS GWAS / eQTL loci (regional colocalization probability (RCP) > 0.2). The SNP with the highest SNP colocalization probability (SCP) for each association is included. * Colocalized loci where the eGene is the closest gene.

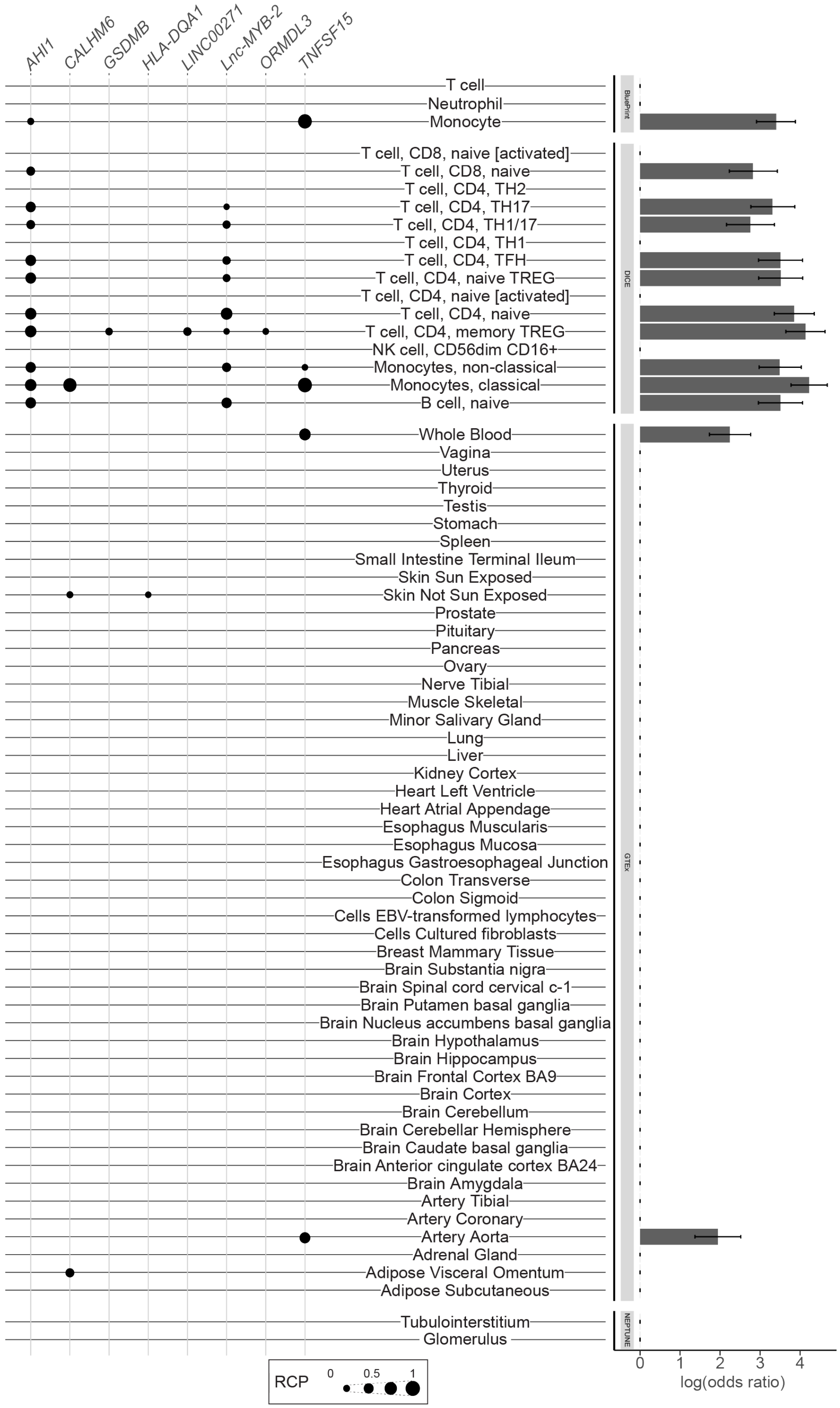

Supplementary Figure 7. Colocalization of SSNS GWAS and all eQTL datasets

Each eQTL data set is labeled with colocalized loci (left) and enrichment estimates (right). The source of each eQTL dataset is labeled on vertical gray bars. Genes with regional colocalization probability (RCP) > 0.2 in at least one tissue/cell are included. pSSNS GWAS loci that colocalized with tissue/cell-type eQTLs are indicated by black dots, with larger dots indicating higher RCP. Enrichment estimates from fastENLOC are based on genome-wide summary statistics from GWAS and include a shrinkage parameter that results in 0 enrichment for multiple tissues/cell-types. Estimates are presented as the logarithm of the odds ratio +/- standard error. logOR=2 ~ OR=7.4, logOR=3 ~ OR=20.1, logOR=4 ~ OR=54.6. eQTL sample sizes: NEPTUNE glomerulus n=240, tubulointerstitial n=311, BLUEPRINT n=200, DICE n=91.

Supplementary Table 7. Credible sets (95%) for genome-wide significant loci in multi-population meta-analysis with open chromatin annotation

RS ID	Position (hg19)	EA	NEA	P-value (MR-MEGA)	PIP	Location	Closest Gene	Kidney open chromatin	Immune open chromatin
rs55730955	2:204585956	A	T	4.27×10^{-10}	0.68	intronic	*CD28*		CD4, CD8
rs1181388	2:204575951	A	G	1.52×10^{-9}	0.18	intronic	*CD28*		
rs3769684	2:204584759	C	T	4.73×10^{-9}	0.07	intronic	*CD28*	LEUK	CD4, CD8, NK
rs4673259	2:204582623	C	T	4.54×10^{-9}	0.06	intronic	*CD28*		CD4, CD34, CMP, GMP, HSC, LMPP, MEP
rs28862935	4:75693465	A	G	1.08×10^{-9}	0.23	intronic	*BTC*		
rs10005089	4:75694606	T	C	2.80×10^{-9}	0.09	intronic	*BTC*	PEC, ENDO, PT1, PT2, PT3, PT, LH, DCT, CNT, PC, ICA, ICB	HSC, LMPP, MEP, MPP
rs6532431	4:75696612	G	T	3.92×10^{-9}	0.06	intronic	*BTC*		
rs28681122	4:75717024	A	G	5.07×10^{-9}	0.05	intronic	*BTC*		
rs4859427	4:75691379	A	G	6.13×10^{-9}	0.05	intronic	*BTC*		
rs28522727	4:75707658	C	T	5.32×10^{-9}	0.04	intronic	*BTC*		
rs28577058	4:75716947	A	G	5.34×10^{-9}	0.04	intronic	*BTC*	POD, MESFIB, CNT, PC, ICA, ICB	
rs60799154	4:75692264	T	C	6.65×10^{-9}	0.04	intronic	*BTC*		
rs28773846	4:75692704	G	T	7.06×10^{-9}	0.04	intronic	*BTC*		
rs10009801	4:75694208	C	T	6.89×10^{-9}	0.03	intronic	*BTC*		
rs28576102	4:75704143	G	A	6.97×10^{-9}	0.03	intronic	*BTC*		
rs72867560	4:75691141	A	G	8.38×10^{-9}	0.03	intronic	*BTC*		ERY
rs72867585	4:75702876	G	A	7.16×10^{-9}	0.03	intronic	*BTC*		
rs28420261	4:75693713	A	C	7.41×10^{-9}	0.03	intronic	*BTC*		
rs28478898	4:75689280	G	C	9.32×10^{-9}	0.03	intronic	*BTC*		
rs28472417	4:75689353	C	T	9.33×10^{-9}	0.03	intronic	*BTC*		
rs59882675	4:75689112	G	A	9.35×10^{-9}	0.03	intronic	*BTC*		
rs10023748	4:75703330	G	T	8.24×10^{-9}	0.03	intronic	*BTC*		
rs28441260	4:75717373	T	C	1.44×10^{-8}	0.02	intronic	*BTC*		
rs6848247	4:75690027	G	T	2.18×10^{-8}	0.01	intronic	*BTC*		
rs28822209	4:75690673	A	G	2.44×10^{-8}	0.01	intronic	*BTC*		
rs58392207	4:75689034	C	T	3.66×10^{-8}	0.01	intronic	*BTC*		
rs2637678	6:116787378	C	T	2.06×10^{-12}	0.74	intergenic	*CALHM6*		
rs2637681	6:116769845	G	T	6.06×10^{-12}	0.24	intergenic	*CALHM6*		
rs7759971	6:135746884	T	C	4.90×10^{-12}	0.40	intronic	*AHI1*		CD34, CLP, CMP, HSC, MPP
rs761357	6:135902599	T	A	3.08×10^{-11}	0.07	intronic	*LINC00271*		
rs11154801	6:135739355	A	C	2.65×10^{-11}	0.07	intronic	*AHI1*		CD34, CMP, HSC, LMPP, MEP, MPP
rs6928977	6:135626348	T	G	4.00×10^{-11}	0.05	intronic	*AHI1*		
rs6908428	6:135793706	G	A	3.45×10^{-11}	0.05	intronic	*AHI1*		CMP
rs11154806	6:135873721	G	T	7.11×10^{-11}	0.03	intronic	*LINC00271*		
rs13197384	6:135818897	A	C	8.13×10^{-11}	0.03	UTR5	*AHI1*	POD, PEC, MESFIB, ENDO, PT1, PT2, PT3, PT, LH, DCT, CNT, PC, ICA, ICB, LEUK	B, CD4, CD8, CD34, CLP, CMP, ERY, GMP, HSC, LMPP, MEP, MONO, MPP, NK
rs2614257	6:135676404	C	T	9.04×10^{-11}	0.02	intronic	*AHI1*		CD34, CMP, MEP, MPP
rs2614266	6:135716532	A	T	1.08×10^{-10}	0.02	intronic	*AHI1*		MPP
rs6914831	6:135639644	C	T	9.52×10^{-11}	0.02	intronic	*AHI1*		
rs4896143	6:135635100	G	C	1.19×10^{-10}	0.02	intronic	*AHI1*		CD8, LMPP, MEP

rs2179780	6:135650266	A	G	1.20×10^{-10}	0.01	intronic	*AHI1*		MEP
rs6570001	6:135651721	G	C	1.61×10^{-10}	0.01	intronic	*AHI1*		B
rs7772681	6:135649013	T	C	1.42×10^{-10}	0.01	intronic	*AHI1*		
rs9647635	6:135841056	C	A	1.69×10^{-10}	0.01	intronic	*LINC00271*		
rs6931735	6:135624811	G	A	1.70×10^{-10}	0.01	intronic	*AHI1*		
rs9385726	6:135836962	T	C	1.72×10^{-10}	0.01	intronic	*LINC00271*		
rs58761508	6:135835901	A	G	1.73×10^{-10}	0.01	intronic	*LINC00271*		
rs9399148	6:135844359	T	A	1.74×10^{-10}	0.01	intronic	*LINC00271*		
rs6935146	6:135627369	T	C	1.79×10^{-10}	0.01	intronic	*AHI1*		CMP
rs7750586	6:135827673	G	A	1.82×10^{-10a}	0.01	intronic	*LINC00271*	CNT, DCT, ENDO, ICA, ICB, LH, MESFIB, PC, PEC, POD, PT1, PT2, PT3, PT, PTKIM1P	B, CD34, CD4T, CD8T, CLP, CMP, GMP, HSC, LMPP, MEP, MPP, NK
rs13208505	6:135893723	T	G	2.10×10^{-10}	0.01	intronic	*LINC00271*		
rs2614255	6:135663581	T	C	1.79×10^{-10}	0.01	intronic	*AHI1*		
rs7759677	6:135909796	C	T	2.20×10^{-10}	0.01	intronic	*LINC00271*	PT1, PT2, PT3, PT	
rs9389286	6:135640965	C	G	3.73×10^{-10}	0.01	intronic	*AHI1*		CD8T
rs4896153	6:135833463	T	A	4.18×10^{-10}	<0.00	intronic	*LINC00271*		
rs2327613	6:135662372	T	C	4.56×10^{-10}	<0.00	intronic	*AHI1*		
rs9321501	6:135641417	C	A	5.00×10^{-10}	<0.00	intronic	*AHI1*		
rs3827780	6:135709760	G	A	6.08×10^{-10}	<0.00	intronic	*AHI1*		
rs2064430	6:135642756	T	C	5.24×10^{-10}	<0.00	intronic	*AHI1*		
rs2246852	6:135691792	G	A	6.28×10^{-10}	<0.00	intronic	*AHI1*		
rs10817678	9:117579457	A	G	5.57×10^{-12}	0.29	intergenic	*TNFSF15*		
rs7848647	9:117569046	C	T	5.99×10^{-12}	0.26	upstream	*TNFSF15*	PT1, PT2, PT3, PT, CNT, PC, ICB, LEUK	B, CD4, CMP, GMP, MPP
rs6478109	9:117568766	G	A	8.12×10^{-12}	0.19	upstream	*TNFSF15*	PEC, ENDO, PT1, PT2, PT3, PT, LH, DCT, CNT, PC, ICA, ICB, LEUK	B, CD4, CD34, CMP, GMP, HSC, LMPP, MPP
rs6478108	9:117558703	T	C	1.37×10^{-11}	0.11	intronic	*TNFSF15*		
rs4263839	9:117566440	G	A	1.59×10^{-11}	0.10	intronic	*TNFSF15*		
rs8062322	16:11092319	A	C	1.61×10^{-10}	0.94	intronic	*CLEC16A*		
rs887864	16:11158885	G	A	3.32×10^{-9}	0.03	intronic	*CLEC16A*		
rs412175	19:36342103	C	T	2.30×10^{-24}	0.95	intronic	*NPHS1*	ENDO, PT2	
rs56117924	19:36334182	A	G	4.99×10^{-23}	0.05	intronic	*NPHS1*		
Conditional Analysis									
rs2256318	6:31381519	A	G	$9.71 \text{ x } 10^{-18}$	.27	intronic	*MICA*		
rs2256026	6:31379141	G	C	$1.28 \text{ x } 10^{-17}$	.21	intronic	*MICA*		
rs2256328	6:31381637	G	C	$1.71 \text{ x } 10^{-17}$	.16	intronic	*MICA*		
rs75918478	6:31453053	A	G	$1.72 \text{ x } 10^{-17}$	.15	intergenic	*MICB*		
rs2857282	6:31380807	A	T	$2.87 \text{ x } 10^{-17}$	.09	intronic	*MICA*		
rs2853982	6:31378751	G	A	$5.64 \text{ x } 10^{-17}$	.05	intronic	*MICA*		
rs2256028	6:31379198	A	C	$6.33 \text{ x } 10^{-17}$	.04	intronic	*MICA*		

The MR-MEGA *P*-value is from test of deviance of full meta-regression model compared to the null model (two-sided). The posterior probability of inclusion (PIP) measures the probability that the SNP is causal, with PIPs for each SNP summing to 0.95. Due to complexity of the region, HLA is excluded from this fine-mapping analysis. EA = effect allele, NEA = non-effect allele.

Kidney cell type codes: POD = podocyte, PEC = parietal epithelial cells, MES-FIB = mesangial and fibroblasts, ENDO = endothelial, PT(1-3) = proximal tubule, PT-KIM1P = proximal tubule with KIM1+ expression, LH =

loop of Henle, DCT = distal convoluted tubule, CNT = connecting tubule, PC = principal cells, ICA = Type A intercalated cells, ICB = Type B intercalated cells, LEUK = leukocytes.

Immune cell type codes: B = CD19+CD20+ B. CD4T, CD8T = CD4+ and CD8+ T, CD34 = CD34+ bone marrow and cord blood, CLP = common lymphoid progenitor, CMP = common myeloid progenitor, Ery = CD71+GPA+ erythroblast, GMP = granulocyte macrophage progenitor, HSC = hematopoietic stem, LMPP = lymphoid-primed multipotent progenitor, MEP = megakaryocyte erythroid progenitor, Mono = CD14+ monocyte, MPP = multipotent progenitor, NK = CD56+ natural killer T.

Supplementary Table 8. Samples used for HLA fine-mapping analysis

GWAS Cohort	Ancestry	n cases	n controls	n total
Nephrovir/EU	European	313	2,508	2,821
US Cohort	European	361	4,309	4,670
	European Total	674	6,817	7,491
US Cohort	African	65	7,335	7,400
Nephrovir/EU	African	44	179	223
	African Total	109	7,514	7,623
US Cohort	South Asian	31	338	369
Indian	South Asian	162	98	260
	South Asian Total	193	436	629
US Cohort	East Asian	16	439	455
Maghrebian	Maghrebian	55	228	283
Admixed American	Admixed American	98	13,248	13,346
Total		1,145	28,682	29,827

Supplementary Table 9. Genome-wide and suggestive loci from ancestry-specific HLA logistic regression and omnibus test

Ancestry	Logistic regression			Omnibus test	
	SNP	P-value	OR [95% CI]	Amino Acid ID	P-value
European	rs28755181	**3.49×10^{-51}**	3.35 [2.86 – 3.92]	AA_DQA1_47_32609219	**6.81×10^{-61}**
African	rs1264705	3.03×10^{-7}	4.64 [2.58 – 8.34]	AA_DQB1_89_32632585	**1.41×10^{-8}**
South Asian	HLA_B*44:03	**9.42×10^{-12}**	2.83 [2.12 - 3.77]	AA_B_199_31323321	**5.64×10^{-10}**
Admixed-American	-	-	-	AA_DQA1_52_32609234	**3.79×10^{-11}**
Maghrebian	rs9273344	2.543×10^{-6}	0.18 [0.09, 0.37]	AA_DQB1_-6_32634305	4.03×10^{-5}

OR [95% CI] = Odds ratio and 95% confidence interval. Alleles reaching genome-wide significance (5×10^{-8}) are in bold. There were no significant associations in the East Asian analysis. Logistic regression analyses included SNPs and classical HLA alleles. P-values are from logistic regression (two-sided) and are unadjusted for multiple testing. Amino Acid ID formatting: Amino acid (AA)_gene_AA position_genomic position. Omnibus P-value from one-sided *F*-test.

Supplementary Note 1:
The strongest pSSNS risk association was SNP rs2856696, between *HLA-DRB1* and *HLA-DQA1* ($P = 2.31 \times 10^{-68}$). The strongest classical HLA allele association was at *DQA1*02* ($P = 2.79 \text{ X } 10^{-59}$). Stepwise conditional analysis identified an independent association near *HLA-DQB1* (rs9273479; $P = 9.97 \text{ X } 10^{-34}$).

Supplementary Table 10. Multi-population HLA logistic regression results

	SNP	EA	*P*-value	OR	Closest Gene
Logistic					
	rs2856696	T	2.31×10^{-68}	2.80	*HLA-DRB1, HLA-DQA1*
	HLA_DQA1*02	T	2.79×10^{-59}	2.49	*HLA-DQA1*
Conditional Logistic					
	rs9273479	T	9.97×10^{-34}	0.49	*HLA-DQB1*
	HLA_DQA1*01	T	3.92×10^{-29}	0.53	*HLA-DQA1*

Results from HLA logistic association test (two-sided, unadjusted for multiple testing). Top overall association and top classical HLA allele are both reported. Results after conditioning on rs2856696 also shown.

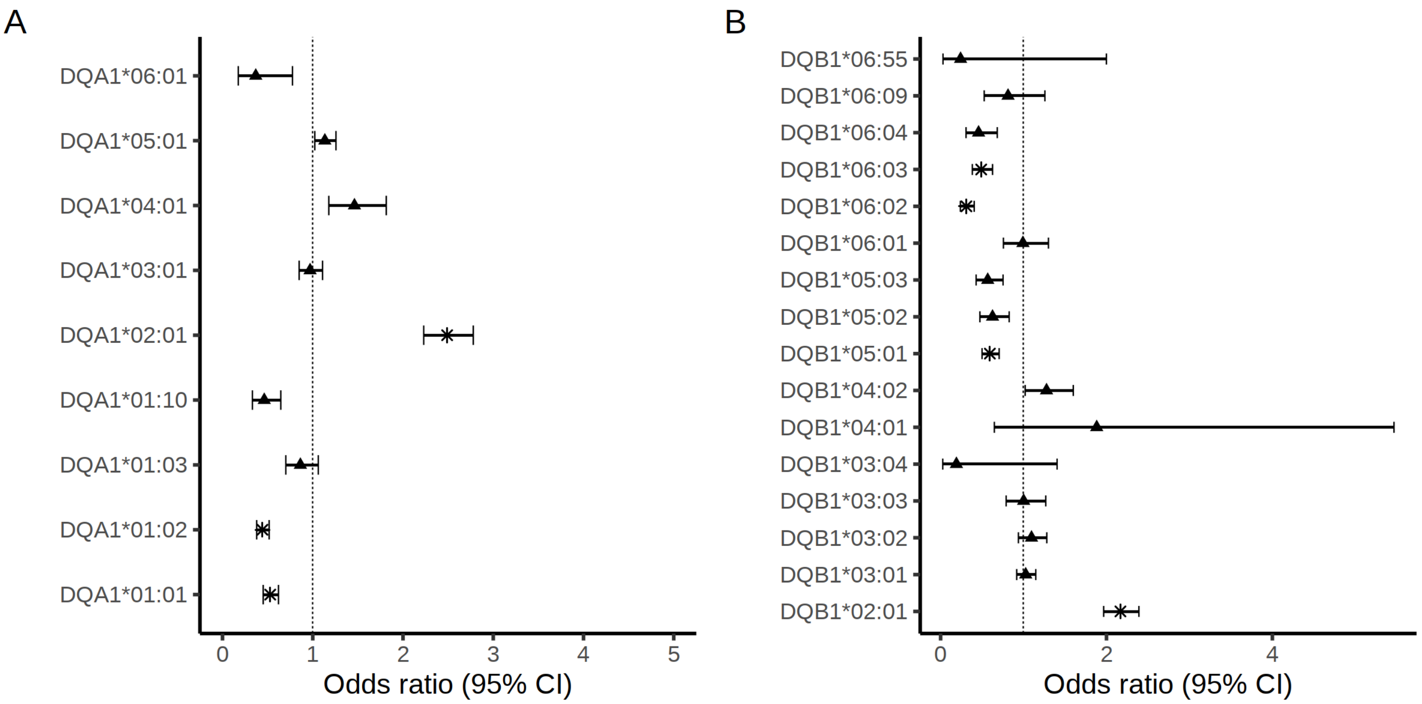

Supplementary Figure 8. HLA classical allele logistic regression results

Forest plot for *HLA-DQA1* (A) and *HLA-DQB1* (B) 4-digit alleles in multi-population analysis (1,145 cases vs. 28,682 controls). Associations reaching genome-wide significance are indicated with *, and nonsignificant with a triangle. Data are presented as odds ratio with 95% confidence interval.

Supplementary Table 11. HLA amino acid omnibus test results

HLA gene & Position	Omnibus P-value	Amino acid	OR [95% CI]	*P*-value
DQA1_47	7.73×10^{-83}			
		cysteine	1.97 [1.75, 2.23]	5.47×10^{-28}
		lysine	3.62 [3.17, 4.14]	5.70×10^{-80}
		glutamine	1.72 [1.47, 2.00]	7.63×10^{-12}
		arginine	ref	
DQA1_52	1.14×10^{-82}			
		serine	0.53 [0.47, 0.59]	1.00×10^{-28}
		histidine	1.92 [1.70, 2.16]	2.55×10^{-27}
		arginine	ref	
DQB1_26*	3.22×10^{-13}			
		tyrosine	0.90 [0.81, 1.00]	0.06
		glycine	0.64 [0.60, 0.73]	4.75×10^{-12}
		leucine	ref	

* Top association after conditioning on DQA1_47 and DQA1_52

Omnibus p-value is from a one-sided *F*-test comparing logistic models with and without amino acid position. Amino acid associations from two-sided logistic regression including all amino acid residues at the given position.

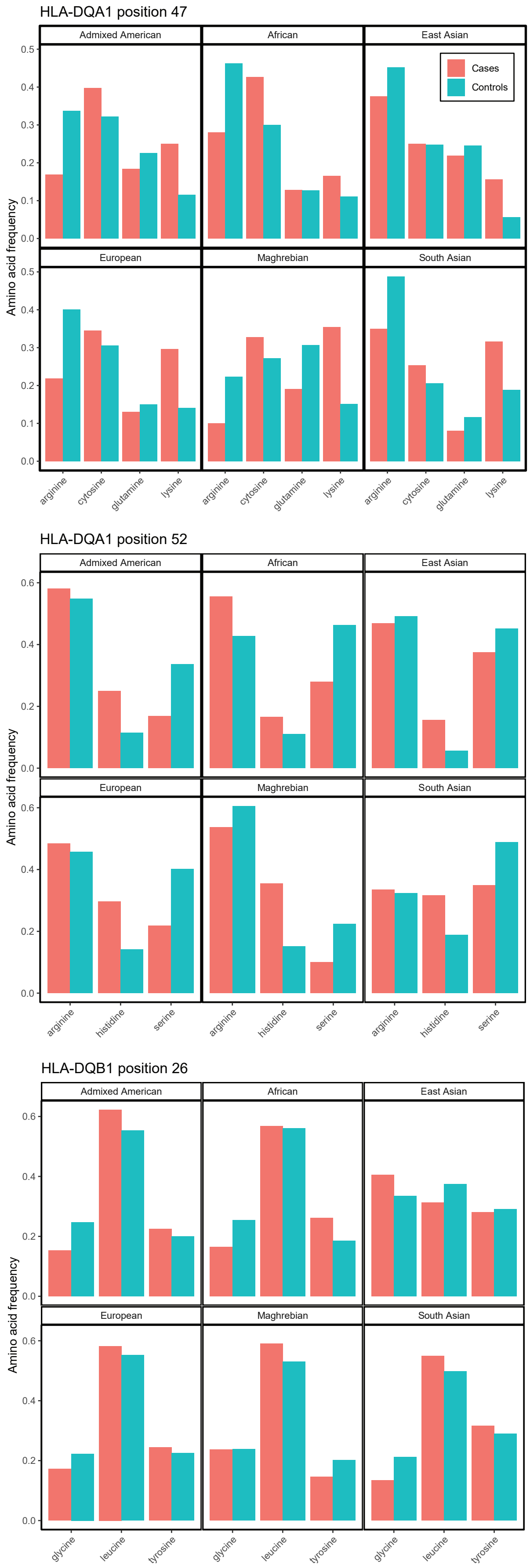

Supplementary Figure 9. Amino acid frequency. Case/control frequency of amino acid residues at positions 47 and 52 of *HLA-DQA1* and 26 of *HLA-DQB1* stratified by ancestry.

Supplementary Table 12. Association of pSSNS polygenic risk score quartiles with sex, relapse pattern, and age of onset

PRS Discovery Population	PRS Range	Model 1: Sex			Model 2: Relapse pattern			Model 3: Age of onset		
		% Male	β_{PRS} (SE)	p-value	% Multiple relapse	β_{PRS} (SE)	p-value	Age of onset	β_{PRS} (SE)	p-value
Multi-population	Q1: [-1.70, -0.35)	0.61	ref	-	0.44	ref	-	6.89 (0.57)	ref	-
	Q2: [-0.35, 0.42)	0.60	0.01 (0.39)	0.98	0.47	0.23 (0.39)	0.55	5.12 (0.45)	-1.44 (0.68)	0.03
	Q3: [0.42, 1.09)	0.55	-0.16 (0.39)	0.68	0.41	-0.17 (0.39)	0.67	5.93 (0.52)	-0.93 (0.68)	0.17
	Q4: [1.09, 3.18]	0.74	0.60 (0.41)	0.14	0.50	0.11 (0.39)	0.79	4.94 (0.40)	-2.05 (0.68)	2.79×10^{-3}
European	Q1: [0.20, 0.67)	0.59	ref	-	0.41	ref	-	6.46 (0.54)	ref	-
	Q2: [0.67, 0.90)	0.55	-0.04 (0.39)	0.92	0.45	0.12 (0.39)	0.76	5.97 (0.51)	-0.68 (0.68)	0.32
	Q3: [0.90, 1.08)	0.59	-0.04 (0.39)	0.92	0.43	0.00 (0.39)	1.00	6.13 (0.52)	-0.60 (0.67)	0.37
	Q4: [1.08, 2.19]	0.78	0.86 (0.43)	0.04	0.53	0.30 (0.39)	0.45	4.34 (0.35)	-2.46 (0.68)	3.48×10^{-4}

Model 1: sex ~ PRS + age of onset + relapse pattern + 4PCs (multiple logistic regression, two-sided)
Model 2: relapse pattern ~ PRS + age of onset + sex + 4PCs (multiple logistic regression, two-sided)
Model 3: age of onset ~ PRS + sex + relapse pattern + 4PCs (multiple linear regression, two-sided)
P-values are not adjusted for multiple testing.

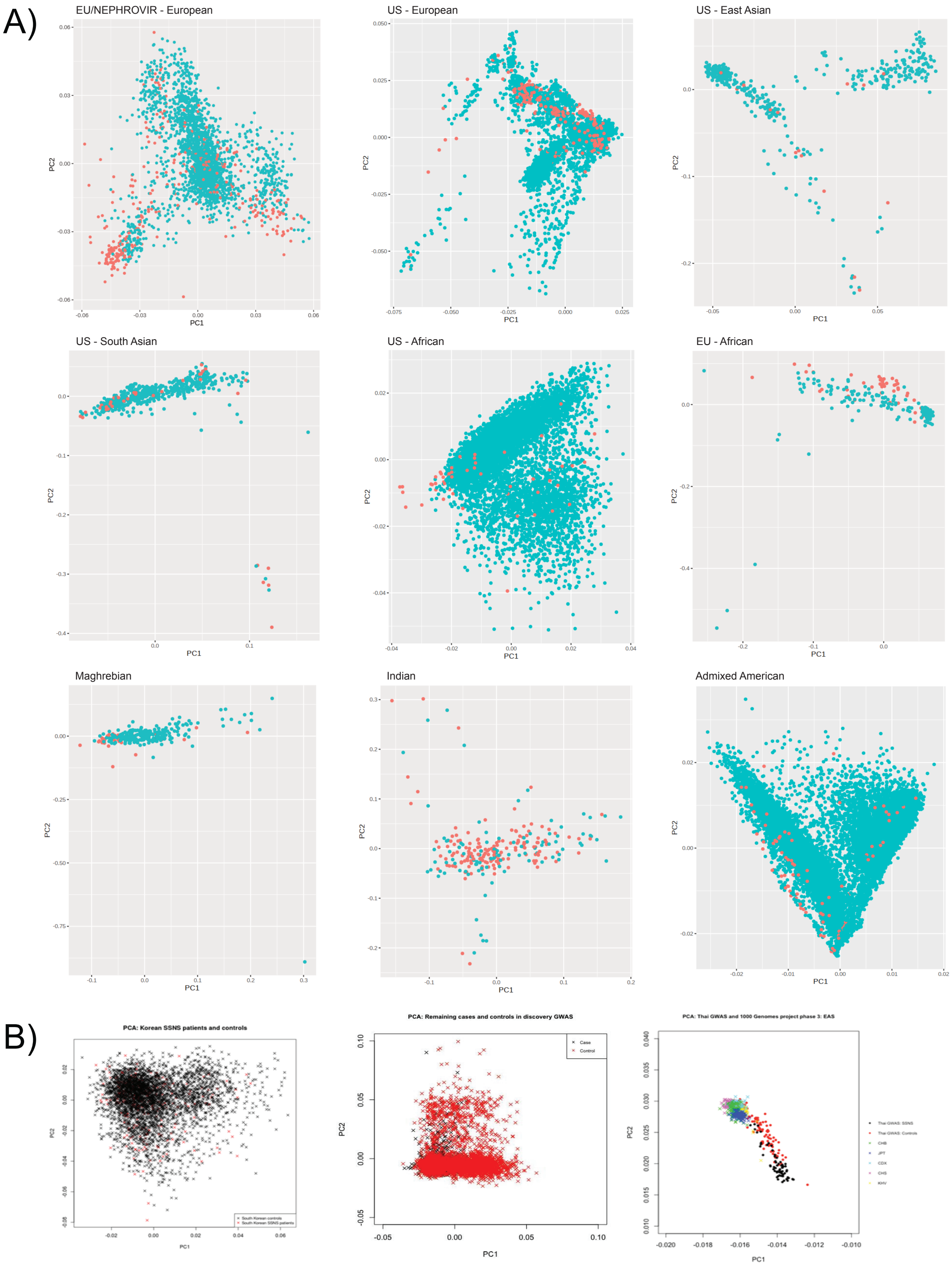

Supplementary Figure 10. Principal component analysis (PC1 vs. PC2) of dataset GWAS cases and controls.

A Principal components for studies with available genotype-level data . Cases are indicated in red; controls are indicated in blue. **B** Principal components from studies with only summary statistics available.

I

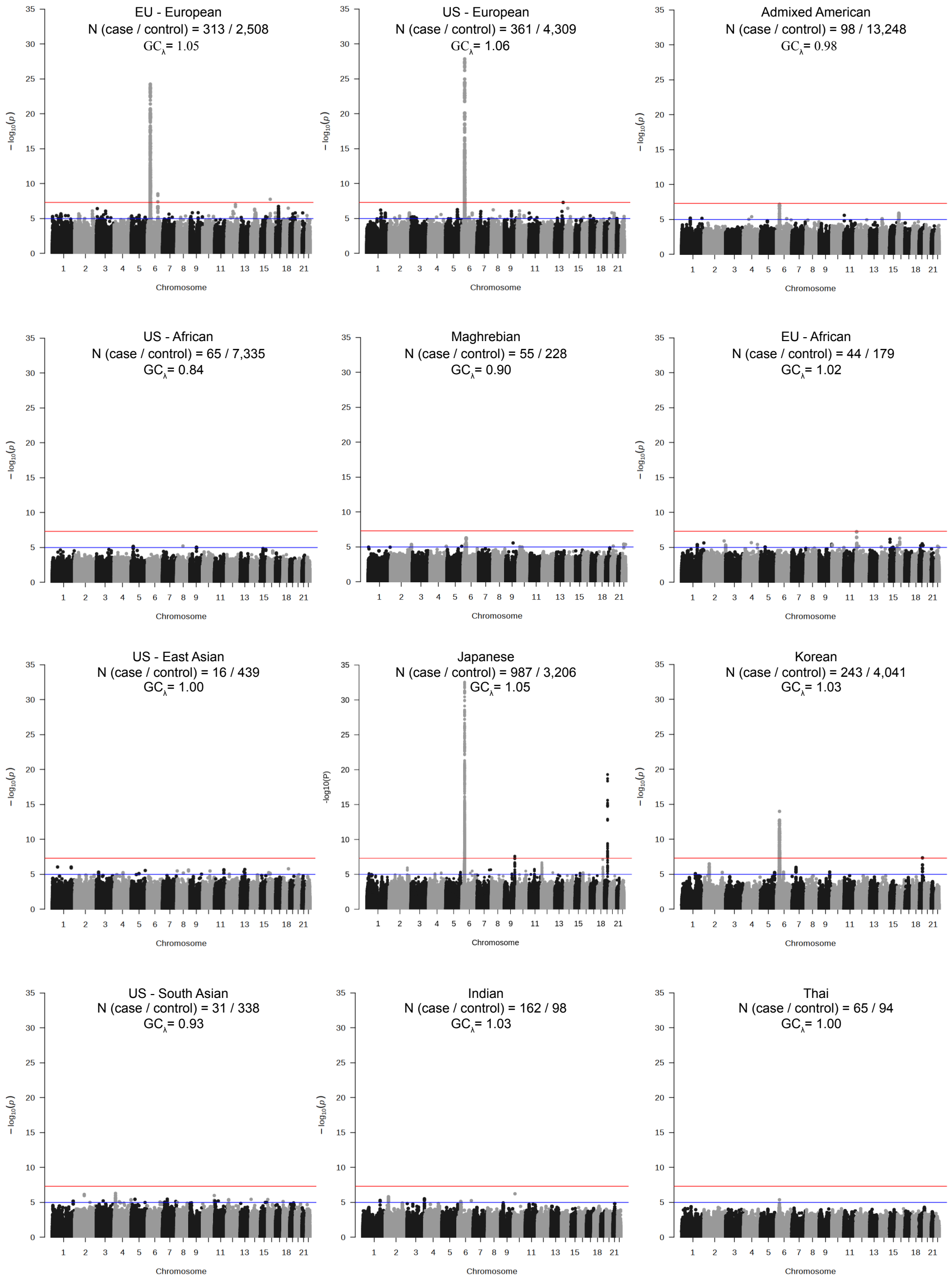

Supplementary Figure 11. GWAS Manhattan plots by cohort.
The red line indicates the genome-wide significance threshold (5×10^{-8}); the blue line is suggestive significance (1×10^{-5}). The number of cases and controls and genomic controls lambda (GCγ) are reported for each dataset. The *P*-values are from logistic regression (two-sided) and are unadjusted for multiple testing. Supplementary Data 1 contains details on models and adjustments.

Supplementary Table 13. Genome-wide significant loci from individual GWAS
All SNP are > 1Mb from each other with $r^2 < 0.1$. We found no genome-wide significant associations in the Thai, EU – African, Maghrebian, Indian, Admixed American, US – East Asian and US – South Asian datasets.
The *P*-values are from logistic regression (two-sided) and are unajusted for multiple testing. Supplementary Data 1 contains details on models and adjustments.

Dataset	Top SNP	Position (hg19)	Nearest Gene	EA	NEA	OR [95% CI]	*P*-Value
EU - European	rs9275205	6:32657560	*HLA-DQB1*	C	T	3.13 [2.52, 3.88]	5.69×10^{-25}
EU - European	rs2637681	6:116769845	*DSE*	G	T	0.54 [0.44, 0.66]	3.01×10^{-9}
EU - European	rs17185137	16:26809795	*C16orf82*	G	A	3.03 [2.06, 4.46]	1.78×10^{-8}
US - European	rs1694129	6:32637743	*HLA-DQB1*	T	G	3.20 [2.61, 3.93]	1.38×10^{-28}
Japanese	rs6901541	6:32442261	*HLA-DRA*	C	T	2.49 [2.15, 2.89]	2.81×10^{-33}
Japanese	rs56117924	19:36334182	*NPHS1*	A	G	1.90 [1.66, 2.18]	4.95×10^{-20}
Japanese	rs117576077	6:31906997	*C2*	T	C	0.13 [0.07, 0.23]	1.85×10^{-11}
Japanese	rs3117032	6:33088592	*HLA-DPB2*	T	C	0.40 [0.30, 0.53]	1.88×10^{-10}
Japanese	rs3095273	6:29566369	*GABBR1*	A	G	0.44 [0.34, 0.58]	6.45×10^{-9}
Japanese	rs6478109	9:117568766	*TNFSF15*	A	G	0.72 [0.64, 0.81]	2.55×10^{-8}
Korean	rs9272518	6:32606446	*HLA-DQA1*	C	G	2.63 [2.06, 3.35]	1.05×10^{-14}
Korean	rs412175	19:36342103	*NPHS1*	C	T	1.83 [1.47, 2.27]	4.66×10^{-8}

Supplementary Table 14: Prediction accuracy for various PRS-CSx hyperparameters to generate multi-population PRS

Gamma-gamma prior*		Global shrinkage parameter**	Prediction accuracy
a	b	ϕ	F-measure
1	0.5	1×10^{-2}	0.327
0.5	0.5	1×10^{-2}	0.327
1.5	0.5	1×10^{-2}	0.324
1	1	1×10^{-2}	0.336
0.5	0.5	1×10^{-4}	0.293
1.5	0.5	1×10^{-4}	0.312
**1**	**1**	$\mathbf{1 \times 10^{-4}}$	**0.350**
1.5	0.5	1×10^{-6}	0.337

*PRS-CSx uses a gamma-gamma hierarchical prior with parameters a and b
** ϕ models the overall sparseness of the genetic architecture. We ranged ϕ from 1×10^{-2} , for highly polygenic traits, and 1×10^{-6}, for less polygenic traits

Supplementary Table 15. Prediction accuracy for various PRS-CS hyperparameters to generate European PRS

Gamma-gamma prior*		Global shrinkage parameter**	Prediction accuracy
a	b	ϕ	F-measure
1	0.5	1 x10^{-2}	0.318
0.5	0.5	1 x10^{-2}	0.314
1.5	0.5	1 x10^{-2}	0.314
1	1	1 x10^{-2}	0.310
1	0.5	1 x10^{-4}	0.310
0.5	0.5	1 x10^{-4}	0.305
1.5	0.5	1 x10^{-4}	0.304
1	1	1 x10^{-4}	0.363
1	0.5	1 x10^{-6}	0.389
0.5	0.5	1 x10^{-6}	0.352
**1.5**	**0.5**	**1 x10^{-6}**	**0.393**
1	1	1 x10^{-6}	0.361

*PRS-CS uses a gamma-gamma hierarchical prior with parameters a and b
** ϕ models the overall sparseness of the genetic architecture
We ranged ϕ from 1 x10^{-2} , for highly polygenic traits, and 1 x10^{-6}, for less polygenic traits

The Research Consortium on Genetics of Childhood Idiopathic Nephrotic Syndrome in Japan

Yoshinori Araki[1], Yoshinobu Nagaoka[1], Takayuki Okamoto[2], Yasuyuki Sato[2], Asako Hayashi[2], Toshiyuki Takahashi[2], Hayato Aoyagi[3], Michihiko Ueno[4], Masanori Nakanishi[5], Nariaki Toita[6], Kimiaki Uetake[7], Norio Kobayashi[8], Shoji Fujita[9], Kazushi Tsuruga[10], Naonori Kumagai[11, 12], Hiroki Kudo[11], Eriko Tanaka[13, 14], Tae Omori[15], Mari Okada[16], Yoshiho Hatai[17], Tomohiro Udagawa[18, 19], Yaeko Motoyoshi[20], Koichi Kamei[21], Masao Ogura[21], Mai Sato[21], Yuji Kano[21, 22], Motoshi Hattori[23], Kenichiro Miura[23], Yutaka Harita[24], Shoichiro Kanda[24], Emi Sawanobori[25], Anna Kobayashi[25], Manabu Kojika[26], Yoko Ohwada[27, 28], Kunimasa Yan[29], Hiroshi Hataya[30], Riku Hamada, Chikako Terano[30], Ryoko Harada[30], Yuko Hamasaki[31], Junya Hashimoto[31], Kenji Ishikura[32], Shuichi Ito[33], Hiroyuki Machida[33], Aya Inaba[33], Takeshi Matsuyama[34], Miwa Goto[35], Masaki Shimizu[36], Kazuhide Ohta[37], Yohei Ikezumi[38, 39], Takeshi Yamada[38], Toshiaki Suzuki[40], Soichi Tamamura[41], Yukiko Mori[41], Yoshihiko Hidaka[42], Daisuke Matsuoka[42], Tatsuya Kinoshita[43], Shunsuke Noda[44], Masashi Kitahara[45], Naoya Fujita[46], Satoshi Hibino[46], Kandai Nozu[47], Tomoko Horinouchi[47], Tomohiko Yamamura[47], China Nagano[47], Shogo Minamikawa[47, 48], Keita Nakanishi[47, 49], Junya Fujimura[47, 50], Nana Sakakibara[47], Yuya Aoto[47], Shinya Ishiko[47], Kazumoto Iijima[51, 52], Ryojiro Tanaka[51], Hiroshi Kaito[51], Kyoko Kanda[51, 53], Yosuke Inaguma[51], Yuya Hashimura[54], Shingo Ishimori[55, 56], Naohiro Kamiyoshi[57], Takayuki Shibano[58], Yasuhiro Takeshima[58], Rika Fujimaru[59], Hiroaki Ueda[59], Akira Ashida[60], Hideki Matsumura[60], Takuo Kubota[61], Taichi Kitaoka[61, 62], Yusuke Okuda[63, 64], Toshihiro Sawai[63], Tomoyuki Sakai[63], Yuko Shima[65], Taketsugu Hama[65], Mikiya Fujieda[66], Masayuki Ishihara[66], Shigeru Itoh[67], Takuma Iwaki[68], Maki Shimizu[69], Koji Nagatani[70], Shoji Kagami[71], Maki Urushihara[71], Yoshitsugu Kaku[72], Manao Nishimura[72], Miwa Yoshino[72], Ken Hatae[73], Maiko Hinokiyama[73], Rie Kuroki[73], Yasufumi Ohtsuka[74], Masafumi Oka[74], Shinji Nishimura[75], Tadashi Sato[76], Seiji Tanaka[77], Ayuko Zaitsu[77], Hitoshi Nakazato[78], Hiroshi Tamura[78], Koichi Nakanishi[79]

1. Department of Pediatrics, National Hospital Organization Hokkaido Medical Center, Sapporo, Japan
2. Department of Pediatrics, Hokkaido University Hospital, Sapporo, Japan
3. Department of Pediatrics, Obihiro Kyokai Hospital, Obihiro, Japan
4. Department of Pediatrics, Nikko Memorial Hospital, Muroran, Japan
5. Department of Pediatrics, Kushiro Red Cross Hospital, Kushiro, Japan
6. Department of Pediatrics, Sapporo Kosei Hospital, Sapporo, Japan
7. Department of Pediatrics, Obihiro Kosei Hospital, Obihiro, Japan
8. Department of Pediatrics, Oji General Hospital, Tomakomai, Japan
9. Department of Pediatrics, Hakodate Goryoukaku Hospital, Hakodate, Japan
10. Department of Pediatrics, Hirosaki University Hospital, Hirosaki, Japan
11. Department of Pediatrics, Tohoku University Graduate School of Medicine, Sendai, Japan
12. Present address: Department of Pediatrics, Fujita Health University, Toyoake, Japan
13. Department of Pediatrics and Developmental biology, Tokyo Medical and Dental University, Tokyo, Japan
14. Present address: Department of Pediatrics, Kyorin University Hospital, Tokyo, Japan
15. Department of Pediatrics, Tokyo Metropolitan Bokutoh Hospital, Tokyo, Japan
16. Musashino Red Cross Hospital, Musashino, Japan
17. Tokyo Bay Urayasu-Ichikawa Medical Center, Urayasu, Japan
18. Tsuchiura Kyodo General Hospital, Tsuchiura, Japan
19. Present address: Department of Pediatrics and Developmental biology, Tokyo Medical and Dental University, Tokyo, Japan
20. Department of Pediatrics, Tokyo Kita Medical Center, Tokyo, Japan
21. Division of Nephrology and Rheumatology, National Center for Child Health and Development, Tokyo, Japan
22. Present address: Department of Pediatrics, Dokkyo Medical University School of Medicine, Mibu, Japan
23. Department of Pediatric Nephrology, Tokyo Women's Medical University, Tokyo, Japan

24. Department of Pediatrics, The University of Tokyo Hospital, Tokyo, Japan
25. Department of Pediatrics, Faculty of Medicine, University of Yamanashi, Chuo, Japan
26. Department of Pediatrics, Fujiyoshida Manucipal Hospital, Fujiyoshida, Japan
27. Department of Pediatrics, Dokkyo Medical University School of Medicine, Mibu, Japan
28. Department of Pediatrics, Tochigi Medical Center Shimostuga, Tochigi, Japan
29. Department of Pediatrics, Kyorin University Hospital, Tokyo, Japan
30. Department of Nephrology, Tokyo Metropolitan Children's Medical Center, Tokyo, Japan
31. Department of Nephrology, Toho University Faculty of Medicine, Tokyo, Japan
32. Department of Pediatrics, Kitasato Univeristy School of Medicine, Sagamihara, Japan
33. Department of Pediatrics, Yokohama City University, Yokohama, Japan
34. Department of Pediatrics, Fussa Hospital, Tokyo, Japan
35. Department of Pediatrics, National Hospital Organization Kofu National Hospital, Kofu, Japan
36. Department of Pediatrics, Kanazawa University Hospital, Kanazawa, Japan
37. Department of Pediatrics, Kanazawa Medical Center, Kanazawa, Japan
38. Department of Pediatrics, Niigata University Medical & Dental Hospital, Niigata, Japan
39. Present address: Department of Pediatrics, Fujita Health University, Toyoake, Japan
40. Department of Pediatrics, National Hospital Organization Niigata National Hospital, Niigata
41. Department of Pediatrics, Japanese Red Cross Fukui Hospital, Fukui, Japan
42. Department of Pediatrics, Shinshu University Hospital, Matsumoto, Japan
43. Department of Pediatrics, Ina Central Hospital, Ina, Japan
44. Department of Pediatrics, Nagano Red Cross Hospital, Nagano, Japan
45. Department of Pediatrics, Matsumoto Medical Center, Matsumoto, Japan
46. Department of Pediatric Nephrology, Aichi Children's Health And Medical Center, Obu, Japan
47. Department of Pediatrics, Kobe University Graduate School of Medicine, Kobe, Japan
48. Present address: Department of General Medicine, Hyogo Prefectural Kobe Children's Hospital, Kobe, Japan
49. Present address: Department of Pediatrics, Saiseikai Hyogoken Hospital, Kobe, Japan
50. Present address: Department of Pediatrics, Kakogawa Central City Hospital, Kakogawa, Japan
51. Department of Nephrology, Hyogo Prefectural Kobe Children's Hospital, Kobe, Japan
52. Department of Advanced Pediatric Medicine, Kobe University Graduate School of Medicine, Kobe, Japan
53. Department of Pediatrics, National Hospital Organization Kobe Medical Center
54. Department of Pediatrics, Takatsuki General Hospital, Takatsuki, Japan
55. Department of Pediatrics, Kakogawa Central City Hospital, Kakogawa, Japan
56. Present address: Department of Pediatrics, Takatsuki General Hospital, Takatsuki, Japan
57. Department of Pediatrics, Himeji Red Cross Hospital, Himeji, Japan
58. Department of Pediatrics, Hyogo College of Medicine, Nishinomiya, Japan
59. Department of Pediatrics, Osaka City General Hospital, Osaka, Japan
60. Department of Pediatrics, Osaka Medical College, Takatsuki, Japan
61. Department of Pediatrics, Osaka University Graduate School of Medicine, Suita, Japan
62. Present address: Department of Pediatrics, Yodogawa Children Hospital, Osaka, Japan
63. Department of Pediatrics, Shiga University of Medical Science, Otsu, Japan
64. Present address: Department of Pediatrics, Kitasato University School of Medicine, Sagamihara, Japan
65. Department of Pediatrics, Wakayama Medical University, Wakayama, Japan
66. Department of Pediatrics, Kochi Medical School, Kochi University, Nankoku, Japan
67. Department of Pediatrics, Kagawa Prefecture Central Hospital, Takamatsu, Japan
68. Department of Pediatrics, Faculty of Medicine, Kagawa University, Kagawa, Japan
69. Department of Pediatrics, Faculty of Medicine, Kagawa University, Kagawa, Japan
70. Department of Pediatrics, Uwajima City Hospital, Uwajima, Japan
71. Department of Pediatrics, Institute of Biomedical Sciences, Tokushima University Graduate School, Tokushima, Japan
72. Department of Nephrology, Fukuoka Children's Hospital, Fukuoka, Japan
73. Department of Pediatrics, Japanese Red Cross Fukuoka Hospital, Fukuoka, Japan
74. Department of Pediatrics, Faculty of Medicine, Saga University, Saga, Japan

75. Department of Pediatrics, Saga-ken Medical Centre Koseikan, Saga, Japan
76. Department of Pediatrics, National Hospital Organization Ureshino Medical Center, Ureshino, Japan
77. Department of Pediatrics and Child Health, Kurume University School of Medicine, Kurume, Japan
78. Department of Pediatrics, Faculty of Life Sciences, Kumamoto University, Kumamoto, Japan
79. Department of Child Health and Welfare (Pediatrics), Graduate School of Medicine, University of the Ryukyus, Nishihara, Japan

Korean Consortium of Hereditary Renal Diseases in Children

Min Hyun Cho[1], Tae-Sun Ha[2], Hee Gyung Kang[3], Il-Soo Ha[3], Ji Hyun Kim[3], Peong Gang Park[3], Kyoung Hee Han[4], Eun Mi Yang[5], Myung Hyun Cho[6], Hae Il Cheong[6]

1. Department of Pediatrics, Kyungpook National University, School of Medicine, Daegu, Korea
2. Department of Pediatrics, Chungbuk National University College of Medicine, Cheongju, Korea
3. Department of Pediatrics, Seoul National University Children’s Hospital, Seoul, Korea
4. Department of Pediatrics, Jeju National University School of Medicine, Jeju, Korea
5. Department of Pediatrics, Chonnam National University Children’s Hospital, Gwangju, Korea
6. Department of Pediatrics, Hallym University Sacred Heart Hospital, Anyang, Korea

Thailand Team

Prayong Vachvanichsanong[1], Kwanchai Pirojsakul[2]

1. Department of Pediatrics, Faculty of Medicine, Prince of Songkla University, Hat-Yai, Songkhla, Thailand
2. Department of Pediatrics, Faculty of Medicine, Ramathibodi Hospital Mahidol University, Bangkok, Thailand
