# Supplementary material for: Multi-population genome-wide association study implicates immune and non-immune factors in pediatric steroid-sensitive nephrotic syndrome
Source: Nat Commun. 2023 Apr 29;14:2481. doi: 10.1038/s41467-023-37985-w (PMC10148875; doi:10.1038/s41467-023-37985-w)
Supplement: Supplementary file 2 — Reporting Summary [file 41467_2023_37985_MOESM2_ESM.pdf]

nature portfolio

Corresponding author(s): Matthew G. Sampson

Last updated by author(s): Mar 20, 2023

Reporting Summary

Nature Portfolio wishes to improve the reproducibility of the work that we publish. This form provides structure for consistency and transparency in reporting. For further information on Nature Portfolio policies, see our Editorial Policies and the Editorial Policy Checklist.

Statistics

For all statistical analyses, confirm that the following items are present in the figure legend, table legend, main text, or Methods section.

n/a	Confirmed	
☐	☒	The exact sample size (*n*) for each experimental group/condition, given as a discrete number and unit of measurement
☒	☐	A statement on whether measurements were taken from distinct samples or whether the same sample was measured repeatedly
☐	☒	The statistical test(s) used AND whether they are one- or two-sided<br>*Only common tests should be described solely by name; describe more complex techniques in the Methods section.*
☐	☒	A description of all covariates tested
☐	☒	A description of any assumptions or corrections, such as tests of normality and adjustment for multiple comparisons
☐	☒	A full description of the statistical parameters including central tendency (e.g. means) or other basic estimates (e.g. regression coefficient) AND variation (e.g. standard deviation) or associated estimates of uncertainty (e.g. confidence intervals)
☐	☒	For null hypothesis testing, the test statistic (e.g. *F*, *t*, *r*) with confidence intervals, effect sizes, degrees of freedom and *P* value noted<br>*Give P values as exact values whenever suitable.*
☐	☒	For Bayesian analysis, information on the choice of priors and Markov chain Monte Carlo settings
☒	☐	For hierarchical and complex designs, identification of the appropriate level for tests and full reporting of outcomes
☐	☒	Estimates of effect sizes (e.g. Cohen's *d*, Pearson's *r*), indicating how they were calculated

Our web collection on statistics for biologists contains articles on many of the points above.

Software and code

Policy information about availability of computer code

Data collection	no software was used
Data analysis	Data QC: Genome studio, Snpflip (https://github.com/biocore-ntnu/snpflip), Plink v1.9, McCarthy tools V4.3, UCSC Liftover, KING, FlashPCA, SAIGE, SHAPEIT, IMPUT2, IMPUTE4 v2.3.1<br>Meta-analysis: METAL v2011-03-25, MR-MEGA v.0.2, GCTA COJO v.1.93.2beta<br>Post-GWAS: LDSC v1.0.1, fastENLOC v1, TORUS, gkmQC v1.0, MACS2 (v2.2.7.1), HLA-TAPAS v2020.05.02. UNIPROT, PyMOL v2.5, SPDBV, DynMut2, Arpeggio, PRS-CSx v7-29-2021<br>Data visualization: qqman v0.1.4, LocusZoom.org, ggplot v3.3.5, R (v3.6.3)

For manuscripts utilizing custom algorithms or software that are central to the research but not yet described in published literature, software must be made available to editors and reviewers. We strongly encourage code deposition in a community repository (e.g. GitHub). See the Nature Portfolio guidelines for submitting code & software for further information.

Data

Policy information about availability of data

All manuscripts must include a data availability statement. This statement should provide the following information, where applicable:

- Accession codes, unique identifiers, or web links for publicly available datasets
- A description of any restrictions on data availability
- For clinical datasets or third party data, please ensure that the statement adheres to our policy

The fixed-effects multi-population summary statistics (METAL) generated by this study have been deposited in the GWAS Catalog [GCST90258619]. The credible sets for significant variants and significant colocalization results generated in this study are provided in the Supplementary file. The raw GWAS data are protected and are not available due to data privacy laws. For HLA modeling, we used the Protein Data Bank (PDB; https://www.rcsb.org/) and UNIPROT database (Uniprot ID: P01909). For colocalization analyses, we used the NEPTUNE cohort (https://www.neptune-study.org/), the unfiltered eQTL results from DICE (https://dice-database.org/downloads), and the BLUEPRINT consortium.

Human research participants

Policy information about studies involving human research participants and Sex and Gender in Research.

Reporting on sex and gender	Only sex was used in this analysis and results apply to both sexes. We used self-reported sex to exclude samples with discordant sex predicted from X-chromosome genotypes. We also used self-reported biologic sex in our polygenic risk score association analysis. This study utilized GWAS summary statistics from multiple research centers and not all individual data, including sex, was available; thus, overall numbers are not reported.
Population characteristics	Overall, we had 2,440 pSSNS cases from 6 different populations: 674 European, 109 African, 1,311 East Asian, 193 South Asian, 55 Maghrebian, and 98 Admixed American. We combined controls collected at each research facility with publicly available datasets for a total of 36,023 controls: 6,817 European, 7,514 African, 7,780 East Asian, 436 South Asian, 228 Maghrebian, and 12,248 Admixed American. All cases had an elevated urine protein to creatinine ratio of a similar magnitude, a low serum albumin of a similar magnitude, a remission of proteinuria with steroids, and pediatric age of onset (< 18 for all studies except those from Columbia University which used < 21). All controls were above 18 years of age.
Recruitment	The inclusion criteria across populations were uniform in requiring (1) an elevated urine protein to creatinine ratio of a similar magnitude, (2) a low serum albumin of a similar magnitude, (3) a remission of proteinuria with steroids, and (4) pediatric age of onset (< 18 for all studies except those from Columbia University which used < 21. Each of these definitions would be recognized as appropriate for SSNS by nephrologists across the globe. Some controls are sourced from publicly available cohorts (PAGE, 1000 Genomes), assuming that, as a rare disease, pSSNS cases were absent within them. If anything, this bias would result in reduced power.
Ethics oversight	This research was conducted with the informed consent of all study participants and had ethical approval from the Boston Children's Hospital IRB.

Note that full information on the approval of the study protocol must also be provided in the manuscript.

Field-specific reporting

Please select the one below that is the best fit for your research. If you are not sure, read the appropriate sections before making your selection.

☒ Life sciences ☐ Behavioural & social sciences ☐ Ecological, evolutionary & environmental sciences

For a reference copy of the document with all sections, see nature.com/documents/nr-reporting-summary-flat.pdf

Life sciences study design

All studies must disclose on these points even when the disclosure is negative.

Sample size	No sample size calculations were performed. Power was maximized by combining all available pSSNS GWAS data representing the largest pSSNS GWAS sample size to date. After quality control, our multi-population meta-analysis of pediatric steroid sensitive nephrotic syndrome included 2,440 cases and 36,023 controls across 12 datasets and 6 populations. We combined previously published data (Debiec et al., JASN, 2018, Jia et al., KI, 2020), newly generated data, and publicly available controls (PAGE, 1000 Genomes). Given previous GWAS of pSSNS of smaller sample sizes, these samples are sufficient to increase genome-wide discoveries.
Data exclusions	Across all 12 datasets, variants and sample were excluded through pre-established routine quality control metrics. SNPs were excluded due to low frequency, significant deviation from Hardy-Weinberg equilibrium, low genotype rate, and low imputation quality. Samples were excluded with low genotype rate and discordant sex. We also removed any related samples and samples that did not overlap with both cases and controls. These filtering criteria provided high confidence SNPs and carefully matched cases and controls.
Replication	All the available pSSNS GWAS data was used to generate this meta-analysis, which represents the largest pSSNS GWAS to data. Genome-wide

Replication	significant SNPs merging from the meta-analysis design are those that are found to have sufficient effect sizes across multiple cohorts. Each association was compared within and across our 12 datasets and 6 populations to exclude spurious results. As such, these represent replication of signals across independent cohorts.
Randomization	Randomization is not applicable to our genetic association tests.
Blinding	Blinding is not applicable to our genetic association tests.

Reporting for specific materials, systems and methods

We require information from authors about some types of materials, experimental systems and methods used in many studies. Here, indicate whether each material, system or method listed is relevant to your study. If you are not sure if a list item applies to your research, read the appropriate section before selecting a response.

Materials & experimental systems

n/a	Involved in the study
☒	☐ Antibodies
☒	☐ Eukaryotic cell lines
☒	☐ Palaeontology and archaeology
☒	☐ Animals and other organisms
☒	☐ Clinical data
☒	☐ Dual use research of concern

Methods

n/a	Involved in the study
☒	☐ ChIP-seq
☒	☐ Flow cytometry
☒	☐ MRI-based neuroimaging
